# Non-animal models for blood–brain barrier permeability evaluation of drug-like compounds

**DOI:** 10.1038/s41598-024-59734-9

**Published:** 2024-04-17

**Authors:** Frederic O. Dehnbostel, Vaibhav A. Dixit, Robert Preissner, Priyanka Banerjee

**Affiliations:** 1 Institute for Physiology, Charité – University Medicine Berlin, 10115 Berlin, Germany; 2 Department of Medicinal Chemistry, Department of Pharmaceuticals, National Institute of Pharmaceutical Education and Research, Guwahati, (NIPER Gu-Wahati), Ministry of Chemicals and Fertilizers, Government of India, Sila Katamur (Halugurisuk), Kamrup, P.O.: Changsari, Guwahati, Assam 781101 India

**Keywords:** Machine learning, CNS, Blood–brain barrier, Permeability, Computational prediction, Model, Central nervous system, CNS drug discovery, Computational models, Cheminformatics

## Abstract

Diseases related to the central nervous system (CNS) are major health concerns and have serious social and economic impacts. Developing new drugs for CNS-related disorders presents a major challenge as it actively involves delivering drugs into the CNS. Therefore, it is imperative to develop in silico methodologies to reliably identify potential lead compounds that can penetrate the blood–brain barrier (BBB) and help to thoroughly understand the role of different physicochemical properties fundamental to the BBB permeation of molecules. In this study, we have analysed the chemical space of the CNS drugs and compared it to the non-CNS-approved drugs. Additionally, we have collected a feature selection dataset from Muehlbacher et al. (J Comput Aided Mol Des 25(12):1095–1106, 2011. 10.1007/s10822-011-9478-1) and an in-house dataset. This information was utilised to design a molecular fingerprint that was used to train machine learning (ML) models. The best-performing models reported in this study achieved accuracies of 0.997 and 0.98, sensitivities of 1.0 and 0.992, specificities of 0.971 and 0.962, MCCs of 0.984 and 0.958, and ROC-AUCs of 0.997 and 0.999 on an imbalanced and a balanced dataset, respectively. They demonstrated overall good accuracies and sensitivities in the blind validation dataset. The reported models can be applied for fast and early screening drug-like molecules with BBB potential. Furthermore, the bbbPythoN package can be used by the research community to both produce the BBB-specific molecular fingerprints and employ the models mentioned earlier for BBB-permeability prediction.

## Introduction

Neurological disorders account for a large and increasing disease burden worldwide^1^. Neurological and psychiatric disorders account for 1.4% of all deaths and are yet poorly treatable^1^. However, they are estimated to account for a remarkable 28% of all years of life lived with disability^2^. Disorders of the central nervous system (CNS) are associated with multiple disease states of significant social and economic impact. The development of new therapeutics for disorders acting on the CNS presents exceptional challenges compared to other diseases. These challenges include incomplete or limited understanding of the molecular mechanisms involved in multifaceted CNS diseases like Alzheimer's disease, and the presence of the blood–brain barrier (BBB) that restricts the delivery of drugs to the brain.

Furthermore, there is an absence of clinically relevant animal models to test new drug-like molecules with reliable outcomes. The BBB is a strict permeation barrier, maintaining brain homeostasis and selectively controlling the passing of nutrients and solutes of the blood into the CNS, thereby representing a bottleneck for therapeutics meant to treat CNS-related diseases^3^. It is important that CNS drugs are able to penetrate this dynamic and selectively protective BBB membrane. The exchange of external and native agents between the blood and CNS is known to be restricted by physicochemical factors like large molecular weight, high topological polar surface area, and a high degree of hydrogen bonding^4^. While exceptions exist, these factors drastically limit the use of peptides, antibodies and polar small molecules as orally administered CNS drugs^4^. Thus, how much ‘free-drug’ is available to bind to the receptors present in the CNS cannot be overstated. Only the concentration of the plasma unbound drug at the site of action is responsible for pharmacological response in vivo^5^. Thus, there is an increasing demand for therapeutics that are able to effectively modulate targets in the CNS. The CNS drug discovery research unarguably is one of the most challenging endeavours in the pharmaceutical industry and academia^5^.

Often, the ratio or log-ratio of the drug-brain concentration to the drug-blood concentration at the steady state indicates a molecule’s BBB permeability potential^6^. Although, in vivo experiments are traditionally considered the most accurate approach to measure a drug’s BBB penetration potential and remain unmatched with respect to sensitivity. Such in vitro or in vivo evaluation of BBB permeability is not only time-intensive but expensive, being limited in throughput and necessitating elaborate set ups when compared with in silico approaches^3–7^. Therefore, it is imperative to develop in silico methodologies to reliably identify potential lead compounds that can penetrate the BBB as well as determine physico-chemical properties fundamental to the BBB permeation of molecules. Especially as almost 100% of large and more than 98% of small molecules do not cross the BBB into the CNS^8^, a fast-screening method that indicates which compounds should be further investigated is indispensable when designing new CNS drugs. It is moreover important to ensure that drugs having targets outside the CNS do not cross the BBB to avoid unwanted side-effects, particularly since several studies found that neurotoxicity poses the third most common adverse drug reaction resulting in the withdrawal of a drug^9^, further increasing the benefit of a high-throughput and reliable in silico model.

Several computational approaches have been developed and applied to predict favourable CNS drug properties, aiding the selection and optimisation of lead compounds^10^. Multiple studies aiming at producing machine learning models capable of reliably predicting blood–brain-barrier (BBB) permeation have been published^11–15^.

Muehlbacher et al.^11^ compiled a dataset from literature, resulting in 202 compounds, 126 positive class and 76 negative class samples. Substrates of the efflux-pump P-Glycoprotein were deleted from the dataset as these are likely to be removed from the CNS. The Random Forest-based model was evaluated using a bootstrap (n = 100) and achieved an internal *Accuracy* of 0.878. No external validation was performed. Castillo-Garit et al.^12^ used a dataset of 497 molecules that was split into a training and a test set consisting of 381 and 116 compounds, respectively. While the training set is composed of 200 positive and 181 negative samples, the composition of the test set was not disclosed. A decision tree-based model was employed that reached an accuracy of 0.879, a *sensitivity* of 0.867, a specificity of 0.897 and a *Matthews correlation coefficient* (MCC) of 0.76 in the external validation. Internal validation was performed using a tenfold cross-validation^12^. Another work, from Gao et al.^14^, used two datasets. The first is based on CNS-activity, consisting of 161 drugs, 76 CNS- active and 85 CNS-inactive compounds. The second dataset contains a total of 213 compounds, 139 positive and 74 negative class instances, which were labeled according to their *log BB* value^14^. Molecules having a *log BB* greater than − 1 were labeled as active, those having a *log BB* smaller than − 1 as inactive. Both chemical descriptors and drug indications and side effects extracted from SIDER^16^ as well as combinations of the aforementioned, were employed for training and evaluation of the classifiers. Independent evaluation of predictions based on clinical phenotypes (side effects and indications) as well as training and cross-validation based on the combination of chemical features (including log P, surface area, molecular weight, and hydrogen bond donors and acceptors) and clinical phenotypes were performed on the first dataset. The second dataset was used for training and cross-validation of the classifiers using only drug side effects and indications. A polynomial kernel Support Vector Machines (SVMs) based on input vectors composed of both chemical descriptors and clinical phenotypes achieved the best test prediction results of an *accuracy* of 0.855 (+/−  0.023), a ROC-AUC of 0.854 (± 0.024) and a *F*1-score of 0.863 (+/−  0.022). It should be mentioned that in case of the classifiers trained and validated with only the chemical descriptors or the combination of chemical descriptors and clinical phenotypes, no external validation was carried out. Wang et al. composed a dataset from four different studies containing a total of 2358 compounds. This was divided into an external validation set of 145 samples, of which 36 were labelled as negative and 109 as positive and a dataset for training and internal validation consisting of 2213 compounds. The second was again split into a training set of 1881 molecules and an internal test set of 332 compounds. As multiple pairs of training and test sets and the splitting techniques employed varied in their approaches, the exact composition of these dataset pairs was not reported. The single best performing model, a SVM using MACCS fingerprints and oversampling method, achieved an *accuracy* of 0.907, a *sensitivity* of 0.917, a *specificity* of 0.873, a G-means of 0.895 and an ROC-AUC of 0.943 in external validation.

Additionally, a consensus model, a three-layer perceptron neural network, was constructed using the predictions of the top five single classifiers—three SVMs and two K-nearest neighbours (KNN)—as input variables. It resulted in an *accuracy* of 0.945, a *sensitivity* of 0.982, a s*pecificity* of 0.833, a G-means of 0.905 and an ROC-AUC of 0.908 in external validation^15^. Thus, several computational models have been developed (quantitative and qualitative) for the prediction of BBB, the overall accuracies of these models range from 75 to 95%. An overview of the different dataset compositions can be found in Table 1.

**Table 1:** Comparison of dataset compositions of other publications and this work.

	Training set (pos./neg.)	Test set(pos./neg.)
Muehlbacher et al.^11^ compiled from literature	126/76	–
Castillo-Garit et al.^12^	200/181	116 (composition not disclosed)
Gao et al.^14^ dataset compiled from multiple works	76/85 (dataset 1) 139/74 (dataset 2)	76/85 (dataset 1)
Wang et al.^15^ dataset compiled from multiple works	1881 (training set; composition not disclosed)	
	332 (internal validation set; composition not disclosed)	
	109/36	
Yuan et al.^17^	1026/248 (Dataset A, Adenot et al.^18^) 1240/352 (Dataset B, Zhao et al.^2^)	257/62 (Dataset A, Adenot et al.^18^) 310/88 (Dataset B Zhao et al.^2^))
Liu et al.^19^	1276/481 (Zhao et al.^2^, Wang et al.^15^)	154/59 (Li et al.^20^)
Alsenan et al.^13^	1803/547 (Wang et al.^15^) 1803/ 1803 (Balanced via SMOTE)	–
This work, bbbPythoN-imb data sets of best performing model	1108/366	325/34
This work, bbbPythoN-bal data sets of best performing model	1537/1754	479/286

In this study, we focus on two machine learning algorithms and two distance metrics, while employing two-dimensional and three-dimensional physicochemical descriptors to improve differentiation of the BBB active and inactive compounds. In addition, two datasets, one balanced and one skewed towards the positive class, were employed to evaluate their influence on the resulting classifiers’ responsiveness. Especially regarding active compounds. Due to BBB-permeating molecules being of higher interest in the context of the development of CNS-active therapeutics, the imbalanced dataset was meant to increase the sensitivities of the resulting models. Moreover, many publications employ datasets favouring the active class (See Table 2). We found that models trained on the imbalanced dataset, though they performed well in internal and external validation, lost in specificities and saw an increase in the rate of False Positive predictions when tested on an additional external validation set. Classifiers based on the balanced dataset performed slightly worse internally and were comparable in external validation. However, their specificity and number of False Positives were much better on the additional external validation. The model performing best on the imbalanced dataset reported in this study achieved an accuracy of 95% and 99%; sensitivity of 98% and 100%; specificity of 97% and 91%; MCC of 0.89 and 0.95; ROC-AUC of 0.98 and 0.99; Cohen’s Kappa of 0.89 and 0.95 on cross-validation and external-validation set respectively. The MI-DSE feature-based fingerprints designed based on the work of Wassermann et al.^21^ and Xue et al.^22^ and employed for training and testing of classifiers in this study have slightly outperformed those models using molecular fingerprints.

**Table 2:** Comparison of the model presented in this study with other published BBB models on external dataset.

Models	Accuracy (%)	Sensitivity (%)	Specificity (%)	AUC-ROC	MCC
This study, bbbPythoN-imb ; RF, 2D + 3D feature fingerprint using 8-bits; Splitting via Kennard-Stone using Mahalanobis distance; External	0.997	1.0	0.971	0.997	0.984
This study, bbbPythoN-bal; RF, 2D + 3D feature fingerprint using 8-bits; Splitting via Kennard-Stone using Mahalanobis distance; External	0.98	0.992	0.962	0.999	0.958
Other models, Yuan et al.^17^; SVM with RBF kernel, 1D, 2D, and 3D descriptors and five different fingerprints; Dataset A External	0.987	1.0	0.932	-	0.958
Others models; Wang et al.^15^; Three layer perceptron consensus model, MACCS fingerprints; SMOTE + ENN oversampling; External	0.966	0.99	0.833	0.919	–
Other models; Liu et al.^19^; Ensemble Method, EState, MACCS, PubChem, FP4, KR, AP2D, FP4C, KRC, and APC2D fingerprints; SMOTE oversampling; External	0.784	0.812	0.712	–	–
Other Models; Alsenan et al.^13^; Recurrent Neural Network, 6394 descriptors and fingerprints; SMOTE oversampling; Internal K-fold validation	0.965	0.949	0.98	0.986	0.931

## Materials and methods

### Data preparation

First we collated a feature selection dataset from the supplementary material of Muehlbacher et al.^11^ and an in-house dataset. Duplicates and intersections were removed with the dataset used for training and testing our models. Resulting in 481 compounds, of which 253 are BBB permeating and 228 do not cross the BBB. This dataset was solely used for the identification of relevant descriptors.

The imbalanced data used to train the models was obtained using an in-house text-mining pipeline via the PubMed query search^23^ and was manually curated by domain experts^24^. Additionally, datasets were obtained and integrated from published literature sources^2,14,17^. The data was standardised by removing noncovalent, inorganic, mixtures and salts. Additionally, those with greater molecular weight were removed (> 1000 daltons). To further divide the compound dataset into binary classes: actives (BBB+) and inactives (BBB− ), we have used Log BB ≥ − 1 and Log BB < − 1, respectively. We have used KNIME analytics platform to standardise the dative bonds and generate canonical SMILES for each chemical structure. Duplicates and ambiguous compounds/contradictory data were removed from the final dataset (as shown in Tables 1 and 2). The total number of unique compounds corresponds to 1875.

The dataset was split into training and testing sets employing the Kennard-Stone algorithm^25,26^ based on the descriptors identified in the feature selection process. The complete set consists of 1842 molecules (compounds for which 3D coordinates or descriptors could not be calculated were removed), 1440 active and 402 inactive, and the training set and external test set contain 1474 and 368 molecules, respectively. As the splitting via the Kennard-Stone is done separately for each combination of descriptors (2D, 3D and 2D + 3D), and distance metric (euclidean and mahalanobis), and one additional time for the Morgan2 fingerprints), overall, seven pairs of training and external test sets were produced.

The balanced dataset was obtained from the B3DB GitHub repository^27^, which consisted of 4956 BBB-permeating and 2852 BBB-non-permeating compounds. As stated in their publication, compounds were cleaned of salts and solvents, and those with heavy metal atoms were removed. Molecules were normalised, and charges were neutralised. The division into classes was performed using the same threshold of a logBB of − 1 as in creating the imbalanced dataset.

The data preprocessing consisted of cleaning the compounds by disconnecting metal atoms, normalising and neutralising the molecules. Also, compounds overlapping with the additional external validation set, assembled from Li et al.^20^, Wang et al.^15^, and Tong et al.^28^, were removed. This led to 4037 active and 2059 inactive remaining molecules. To balance the dataset, all inactive compounds were retained, while an equally sized subset of the active molecules was randomly sampled. The complete set consists of 2059 compounds per class.

The resulting dataset was also split via the Kennard-Stone algorithm, producing a training set containing 3291 compounds (1537 BBB+, and 1754 BBB−), and a test set containing 765 compounds (479 BBB+, and 286 BBB−). This was only done once for the set of descriptors performing best on the imbalanced dataset.

Additionally, an extra external validation set was collated from the works of Li et al.^20^, Wang et al.^15^, and Tong et al.^28^. The dataset was preprocessed and balanced in accordance with the B3DB dataset.

It consists of 802 BBB-permeating and 810 non-BBB-permeating compounds.

One further external (blind) validation data set (marketed CNS drugs) was used for an additional sensitivity evaluation of the classifiers. It contains 143 FDA-approved Central Nervous System drugs, i.e.,there are no inactive compounds. These drugs are labeled based on them affecting the CNS; this however does not necessarily coincide with them being able to permeate the BBB. Exceptions may be prodrugs like Fesoteodine, or drugs interacting with pathways outside the CNS, that act on the latter.

### Physicochemical properties of CNS drugs

**Figure 1:**
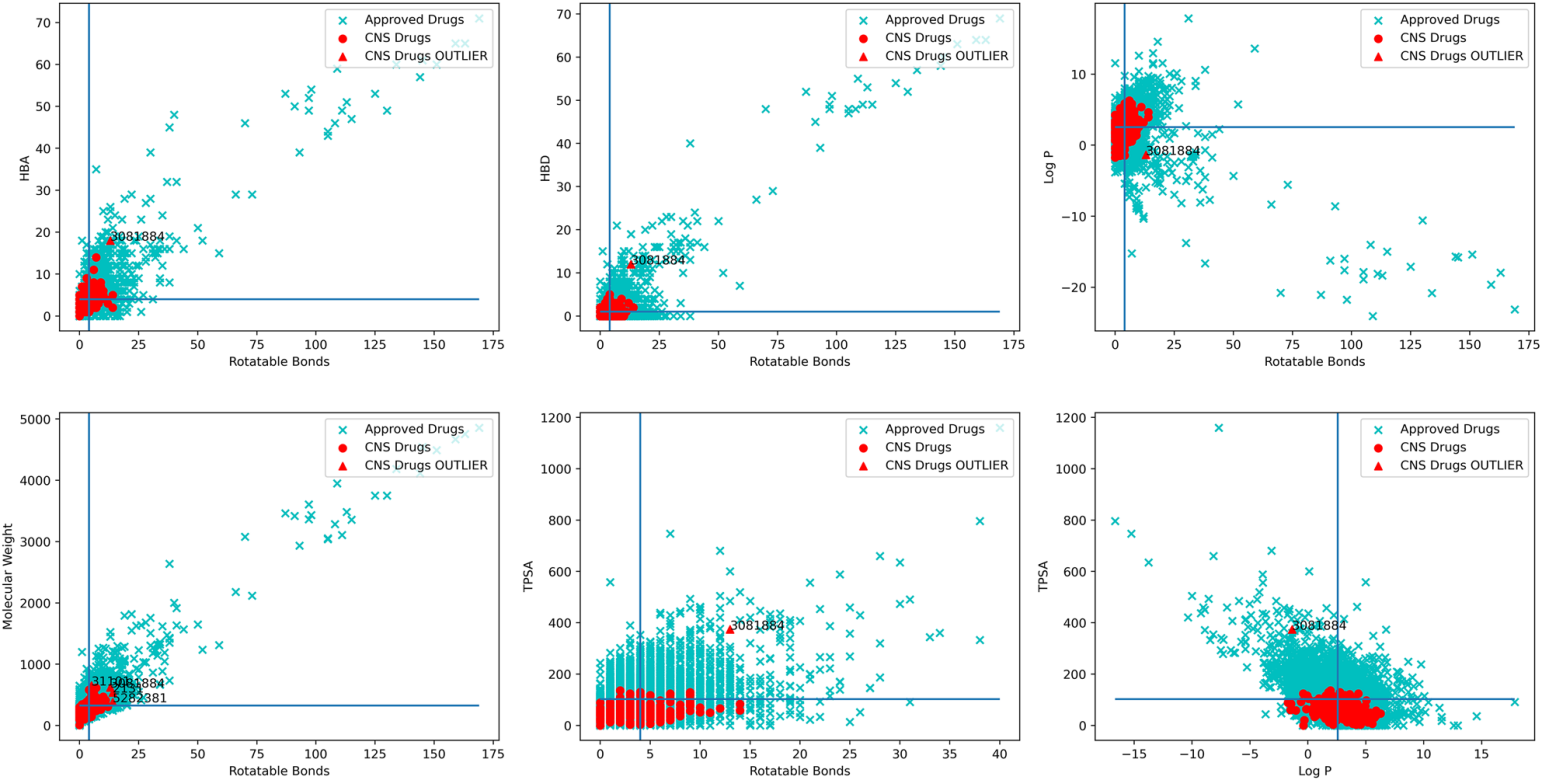
The figure above shows scatterplots of six combinations of the descriptors rotatable bonds, H-bond acceptors, H-bond donors, Log P, molecular weight and TPSA, and displays a clear conservation of CNS drugs around specific values of the aforementioned descriptors. The blue and black lines correspond to the median of the respective descriptor.

Several studies have been published in the literature to understand the physicochemical properties needed to facilitate a drug’s BBB permeability. Using such analysis, many researchers have tried to define the characteristics of successful and unsuccessful CNS drugs^29–31^. It is prudent to mention that the dataset choice greatly influences the conclusions drawn from these analyses and can be a subject of discussion regarding the best feature for evaluating drug-brain exposure. In this study, we curated a dataset of 143 approved CNS drugs on the market and compared these with other approved (non-CNS) drugs^32^. The reason we limited our analysis to only market CNS drugs is that it helps us to get an insight not only into the CNS-related properties but also into overall ADMET (adsorption, distribution, metabolism, excretion and toxicity) properties associated with a successful CNS drug candidate. The molecular flexibility can be expressed in terms of the number of rotatable bonds^33^. Most successful CNS drugs tend to have less than eight rotatable bonds compared to other approved drugs, as shown in Fig. 1. The topological polar surface area (TPSA) is a commonly used metric for optimising a drug's ability to permeate cell membranes. TPSA of CNS drugs tends to be considerably lower than of non-CNS drugs; TPSA < 90 Å^2^ as a cut-off for an optimal CNS drug has been proposed by Waterbeemd et al.^31^ based on a similar analysis.

Additionally, CNS drugs have significantly fewer numbers of hydrogen-bond donors (HBD) and hydrogen-bond acceptors (HBA) compared to non-CNS drugs. Pajouhesh and Lenz proposed the following frequently quoted guidelines for successful CNS drug candidates: H-bond donors < 3; H-bond acceptors < 7; and total H-bonds < 8.8^33^. Lipophilicity was reported as the first molecular descriptor to be identified as an important descriptor involved in the CNS drug delivery^5^. It is now widely accepted that increased lipophilicity is also accompanied by increased risk of poor solubility and metabolic stability, as well as a greater probability of non-specific, off-target activities with related toxicological outcomes^34^. Permeability is a principal prerequisite for CNS penetration; high permeability is critical for acute treatments that need fast-acting drugs, e.g., anaesthetics or analgesics for acute pain. It is less important for chronic administration schedules, where even poor permeants can elicit significant pharmacological effects^35^. It is also observed that CNS drugs tend to be smaller in size compared to non-CNS-approved drugs (see Fig. 1). The low molecular weight of CNS drugs also benefits other critical parameters like rotatable bonds, log P and TPSA.

### Molecular descriptors and feature selection

Descriptors were calculated via the Mordred descriptor calculator^36^, overall covering 1613 two-dimensional (2D) and 213 three-dimensional (3D) descriptors.

**Figure 2:**
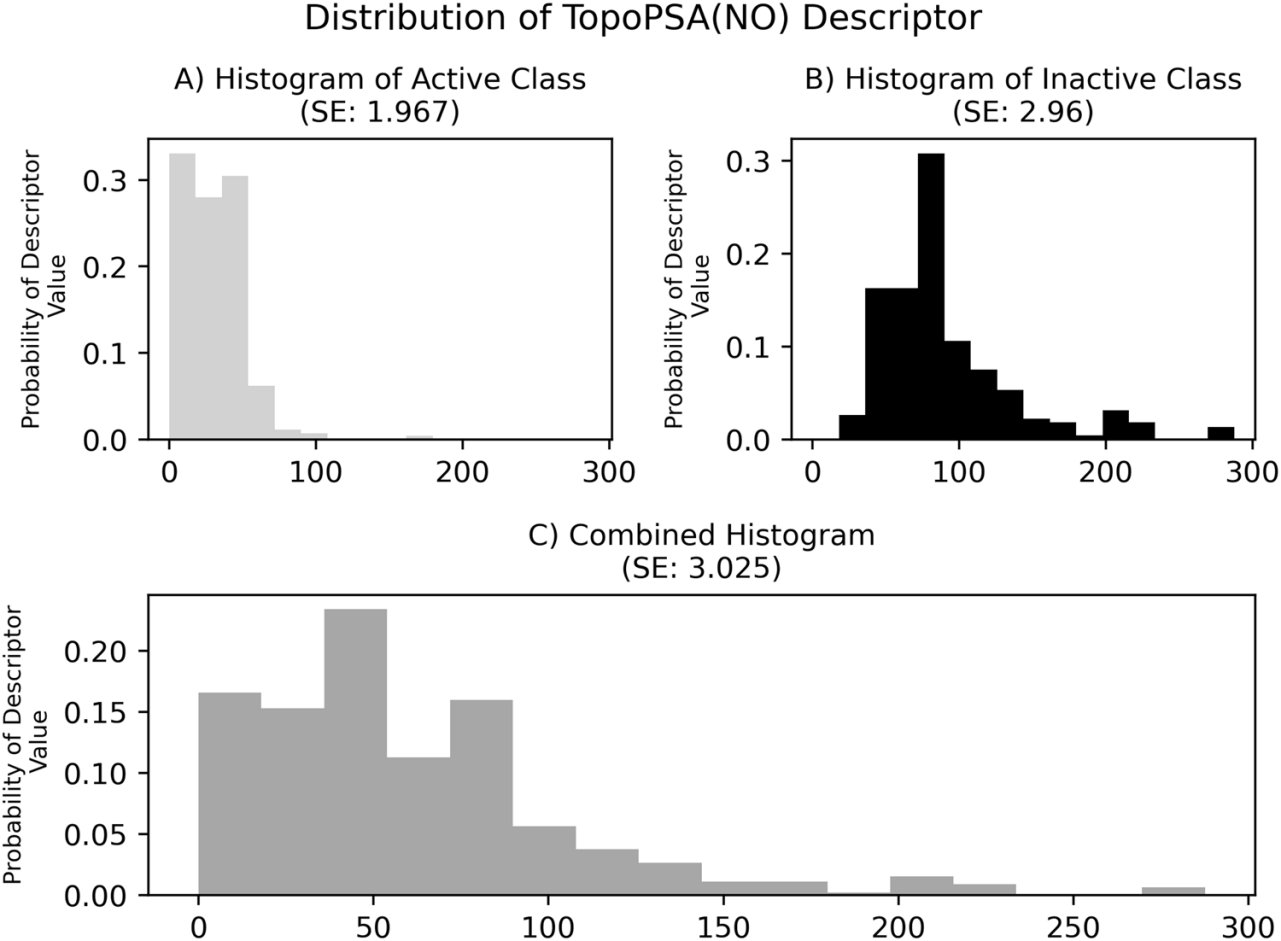
An example of histograms resulting from binning of TopoPSA(NO) values for the class-wise split of the dataset as well as the combined histogram, in which each bins probability is the mean of the respective active and inactive class bins. As observable from the histograms and the given SE, the information content of a descriptor is larger for a wide and equal distribution of values.

Feature selection was performed using the Mutual Information Differential Shannon Entropy (MI-DSE) introduced by Wassermann et al.^21^. The MI-DSE scores a descriptor's capability to discriminate between classes based on its respective value. It is founded on the Shannon Entropy (SE)^37^ and the comparison of feature value distributions between classes using the division of the feature value range into equidistant bins and the allocation of descriptor values into these bins. It is calculated via:

1 $$MI - DSE = H_{AB} \left( D \right) - \frac{{H_{A} \left( D \right) + H_{B} \left( D \right)}}{2}$$

where H_A_(D) is the SE of descriptor D in class A, H_B_(D) is the SE of descriptor D in class B and H_AB_(D) is the SE of descriptor D of the combined distribution (as shown in Fig. 2).

Because of its formulation the MI-DSE is unaffected by a dataset’s class imbalance, which it was specifically designed for. It is moreover normalised to the range between 0 and 1, 1 indicating perfect disjointness and 0 suggesting the identity of the value distributions between classes.

Descriptors were chosen based on their MI-DSE score and the Pearson Correlation Coefficient (PCC). For all pairs of descriptors, the PCC was calculated, and in case of a PCC equal to or greater than 0.8, the descriptor with the higher MI-DSE score was retained, whereas the other was dropped. A maximum of fifty descriptors were selected per set (2D, 3D and 2D + 3D), with a MI-DSE of at least 0.1.

### Structural keys

If feature selection is performed for 2D descriptors an additional feature selection of structural key features is performed. In the case of 2D descriptor calculation using the Mordred descriptor calculator, NaN values occur if a feature represents a structural key or is based on a specific structure and the respective structure is not present in a molecule. The 2D descriptor MDEO-11 for example, describes the molecular distance edge between all primary oxygens. If no pair of primary oxygen atoms is present in the molecule, the MDEO-11 descriptor cannot be calculated, and a NaN value is returned by the Mordred calculator, meaning that a NaN value indicates the absence of the structure described by the feature. The same is true for all 2D descriptors based on specific molecular structures. Of all these structure-based 2D features the relative frequency of NaN values, i.e., the absence of the specific structure, is calculated for both classes separately. The difference of the computed relative frequencies between classes is calculated for each descriptor. Those descriptors having a discrepancy of at least 0.2 are added to the list of structural key features. In the case of E-state descriptors, both the minimal and maximal E-state values of specific atom types—for example, tsC meaning carbon atoms having a triple and a single bond with other atoms—are described by individual descriptors, like MINtsC and MAXtsC. Because both return NaN values if the specified atom type is not present in a molecule, meaning that both contain the same information, only the feature selection retains the maximal E-state value features. The same is true for all other structure-based 2D descriptors having a MAX and MIN instance. The descriptors computing the sum of E-state values of a specified atom type do not return NaN values but a value of zero if no atoms of the given type are part of a compound, so they are not considered in the selection of structural features.

It should be mentioned that in the *Handbook of Molecular Descriptors* it is stated that Molecular Distance-Edge descriptors are “*set at zero by definition if no atom pairs with types s and t are present in the molecular graph*”^38^ [p. 116], where *s* and *t* denote the type of atom-like primary, secondary etc. Nevertheless, in the case of the Mordred descriptor calculator, a NaN is returned if no atom pairs of the given types are present in the molecule.

Because 3D descriptors are not based on the existence or absence of certain structures but on general attributes of the complete molecule, for example, Topological Polar Surface Area, a NaN is only caused by a failure while computing the descriptors and not by missing structures.

### Circular fingerprint

The Morgan fingerprints correspond to circular fingerprints based on the physicochemical properties of the atoms the compound under consideration consists of. An identifier is assigned to each atom encoding its physico-chemical properties along with information regarding its neighbours. In an iterative process, the radius defining an atom’s neighbourhood is increased, producing new values that are then hashed into a new identifier of the respective atom until the specified radius is reached. Finally, the identifiers of all iterations are combined into an array that is used to create the bit fingerprint.

## Methods

### Kennard-Stone: dataset splitting

The Kennard-Stone algorithm (KS) for dataset splitting was introduced in “Computer Aided Design of Experiments”^25^ and was augmented for this study based on “A Modified Kennard-Stone Algorithm for Optimal Division of Data for Developing Artificial Neural Network Models”^26^.

**Figure 3:**
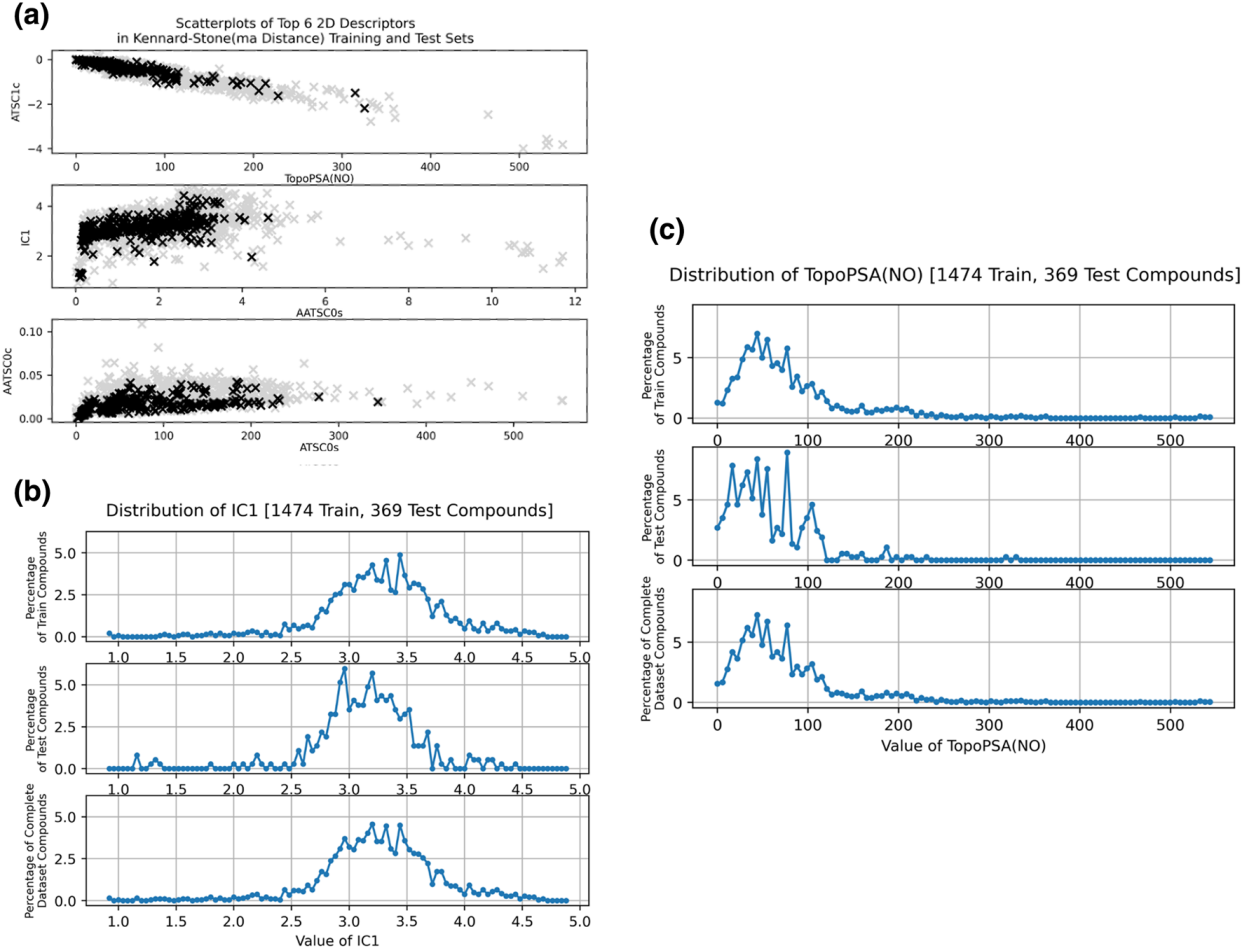
Left: Scatterplot of Top 6 descriptors, black crosses correspond to test set, while gray crosses correspond to training set instances. Right: Distribution of TopoPSA(NO) and IC1 descriptors in training, test and complete dataset. Shows a strong similarity between value distributions of training and complete dataset.

It is designed to split a given dataset into training and test sets, in such a way that the training set covers the complete feature range and reproduces the feature space of the original dataset, which in this case corresponds to the descriptor range and chemical space projected by the dataset compounds (see Fig. 3). To obtain such a subset mirroring the chemical space of the original dataset, first two feature vectors having the maximal distance between each other are selected as initial training set samples. In the following iterative process, a feature vector is appended to the training set if its distance to the nearest training sample is the maximum of all distances between candidate samples and their respective nearest training samples. This is repeated until the specified size of the training set is reached. All remaining feature vectors are assigned to the test set.

As the Mahalanobis distance (MD) has been shown to be superior, particularly in outlier detection, to the Euclidean distance (ED)^39^ and the KS employing the MD outperformed other splitting techniques, including the original KS using the ED, showcased in “A Modified Kennard-Stone Algorithm for Optimal Division of Data for Developing Artificial Neural Network Models”^26^, KS based dataset splitting was performed applying both ED and MD. In case Morgan2 fingerprints were utilised as input vectors, the distance metric was defined as 1-Tanimoto coefficient (TC)^40^, since the TC is a measure of similarity between bit fingerprints.

### Property-based fingerprint design

MI-DSE fingerprints created in this study encode the specific descriptor values of a given molecule into bit strings similar to the encoding scheme described in “Design and Evaluation of a Molecular Fingerprint Involving the Transformation of Property Descriptor Values into a Binary Classification Scheme”^22^. Different grades of precision were used to encode descriptor values (1, 4 and 8 bits), where a feature value encoded by one bit will result in the respective bit being set to 1, if it is equal to or greater than the median of the specific descriptor in the training set and otherwise set to 0.

Provided that features are encoded via four or eight bits the range of the descriptor under consideration in the training set is divided into five or nine bins by four or eight boundaries, which are calculated by:

2 $$bound_{i} = val_{\min } + i*\frac{{val_{range} }}{n},$$

where i is the index of the boundary beginning with 0, val_min_ is the minimum value of the considered descriptor in the training dataset, val_range_ corresponds to the value range of the feature under consideration in the training dataset and n is the number of bits used to encode the descriptor value.

After these boundaries are computed, they are employed in the creation of fingerprints for both training and test set compounds. For each descriptor of a given molecule, the value is compared with the boundaries: if it is less than or equal to the first boundary, it is assigned to the 0-th bin, if it is greater than the last boundary, it is assigned to the fifth or ninth bin. Otherwise, the descriptor value is assigned to the i + 1-th bin if it is greater than the i-th and smaller than or equal to the i + 1-th boundary, where i ranges from zero to three or zero to seven.

By setting the first j bits corresponding to the descriptor to one and the remaining to zero its value is encoded into a bit-string, where j equals the index of the bin the descriptor value was allocated to and is either 0, i + 1, 4 or 8. A fingerprint is produced by concatenating these encoded descriptor values.

**Figure 4:**
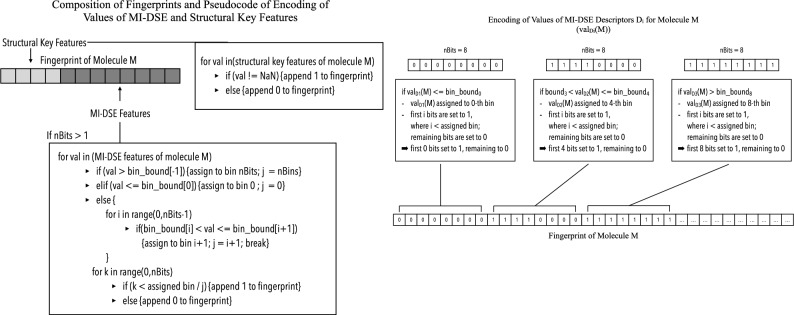
Fingerprint Creation: Pseudocode showing encoding of descriptor values.

When the underlying set of features includes 2D descriptors, the aforementioned structural keys are additionally encoded in the first bit positions as 0, in case of absence, and 1, in case of presence (as shown in Fig. 4).

### Machine learning models

Random Forest (RF) as well as Support Vector Machine (SVM) classifiers, having either a radial basis function (RBF) or polynomial kernel, were constructed and employed in external predictions in this study. The classifiers are part of the scikit-learn python library^41^. As there are 6 distinct pairs of training and test sets, one for each combination of descriptor dimension (2D, 2D + 3D, 3D) and distance metric utilised in dataset splitting (Euclidean and Mahalanobis), in addition to three types of input vectors, each using a different number of bits to encode descriptor values (1, 4, 8), as well as the three classifiers, 54 models were constructed based on our own fingerprints. Moreover, three models were created employing Morgan2 fingerprints, resulting in an overall 57 models.

A Random Forest classifier is composed of multiple decision trees of a specified depth. Each tree is created using a different randomly chosen subset of training set samples. Non-leave nodes are produced by selecting the feature which provides the most homogeneous binary split from a randomly defined subset of features. The quality of each split is measured by a previously specified function. Predictions by the RF are performed via a majority vote^42^.

The principle of SVMs consists of mapping the given training input vectors into a higher dimensional space than the original vectors to be able to construct a hyperplane, also called the decision surface, separating the classes by a maximal margin. Support vectors are those training samples on which the construction of the hyperplane is based, where only a small number of these relative to the total number of training vectors are necessary. Unknown samples are classified by applying a kernel function to calculate their similarity to the projected support vectors, whereby their position in the higher dimensional space relative to the constructed hyperplane can be deduced without actually projecting them into this space.

A decision function returns either a negative or positive value depending on this relative position of new samples^43^.

Hyperparameters were optimised using the exhaustive Grid Search of the scikit-learn python library^41^ prior to training. The Hyperparameters of the RF consist of *max_depth*, *n_estimators*, *max_features*, *class_weight* and *criterion*, which correspond to the depth of each decision tree, the number of trees the RF is composed of, the number of features investigated for the best split in each tree, the weights of each class and the function used to evaluate a split, respectively. In the case of the balanced dataset *max_depth* and *n_estimators* parameters ranged between 200 and 900 and 100 and 1000, both in steps of 100, while the class weights were precomputed using the *compute_class_weight* function of scikit-learn with the *class_weight* parameter set as “balanced”, whereby the weights are defined as $\frac{n\_samples}{n\_classes*class\_count}$.

*max_features* were set to “auto”, defined as $\sqrt{n\_features}$, and as *criterion* both “gini” and “entropy” were supplied.

The best performing imbalanced dataset RF, which is based on the 2D + 3D MI-DSE fingerprints using 8 bits per descriptor as well as the training and test set pair created by the KS utilizing the Mahalanobis distance, employs 100 decision trees each having a depth of 400 in addition to the “Gini” split-evaluation scheme.

The parameter grid for the balanced dataset RF was defined as follows. *max_depth* was chosen from 100, 500, 1000, 1500 and “None”, *n_estimators* from 100, 500, 1500, and 1500. *max_features* were selected from "sqrt", "log2", and “None” and the *criterion* from "gini", "entropy", and "log_loss".

In both above cases “None” corresponds to no limitation. The best performing RF employed 500 *n_estimators*, a *max_depth* of 500, “sqrt” *max_features*, and “entropy” as criterion.

In case of the SVMs *C*, *gamma* and *degree* are the hyperparameters modifying the respective kernel functions, where the latter is only used for RBF kernels. *C* is a penalty parameter balancing the misclassification of training samples and smoothness of the decision surface, which separates the projected feature vectors by class. The *gamma* defines the reach of each support vector, where a high value results in a small radius and a low value corresponds to a large radius of influence, meaning the lower *gamma* is chosen the more distant can a new input vector be from a support vector to be still influenced by it in its classification. *Degree* defines the degree of a polynomial kernel function.

In the case of the imbalanced dataset, the *C* values passed to the Grid Search ranged from 0.7 to 1.2 and *gamma* values ranged from 0.1 to 0.4, both in 0.1 steps. These ranges were chosen after preliminary testing. Whereas *degree* had a value between 1 and 5. The best performing SVM using a polynomial kernel, based on 2D fingerprints encoding descriptor values via 8 bits and the training and test set created by the KS employing the Mahalanobis distance, was created with a *C* equal to 0.7, a *gamma* of 0.1 and a *degree* of 5. The best performing SVM using an RBF kernel, based on 2D + 3D fingerprints encoding descriptor values via 8 bits and the training and test set created by the KS employing the Mahalanobis distance, was created with a *C* of 1.0 and a *gamma* of 0.1.

For the balanced dataset, values ranged from 10^–4^ to 10^4^ and from 10^–4^ to 100, both increasing logarithmically for *C* and *gamma*, respectively. The *degree* was chosen from values of 1 to 6. The best-performing polynomial kernel SVM employed a *C* of 1, a *gamma* of 0.01, and a *degree* of 5. While the best-performing RBF kernel SVM used a *C* 100, and a *gamma* of 0.01.

All models trained and internally validated on the balanced dataset used 2D + 3D MI-DSE fingerprints with an 8-bit feature encoding. The corresponding training and test set pair was produced via the Mahalanobis distance KS.

## Model performance

Multiple metrics were employed in this work to evaluate the performance of each individual classifier. As each metric captures a different aspect of the model performance and has its advantages and disadvantages, it is sensible to use differing metrics to establish knowledge concerning the abilities of a trained model. Following, the metrics used in this work as well as their formulas and characteristics, will be described.

Sensitivity describes the rate of true positives (TP), positive samples that were correctly predicted as positives. While Specificity measures the rate of true negatives (TN), negative samples that were correctly predicted as negatives. As both only measure the predictive accuracy of the model for one activity class, it is sensible to employ them together. Both have a value range of 0 to 1.

3 $$Sensitivity = \frac{TP}{{TP + FN}},$$

where FN is the number of false negatives—samples wrongly predicted as negatives.

4 $$Specificity = \frac{TN}{{TN + FP}}$$

where FP is the number of false positives—samples wrongly predicted as positives.

Accuracy is the ratio between correctly predicted data instances and the total number of data instances that were predicted. In the case of an imbalanced dataset, this metric loses its validity with the increase of disparity between the number of data instances per class. This is because a model's predictive ability will be greater for the majority class, and the influence of minority class predictions on the resulting metric will decrease with the increase of inequality between classes. Moreover, a model will tend to predict all samples it is presented with as majority class instances if the difference in size between classes is sufficiently large, as the overall penalty it is exposed to while training will be negligible^44^.

As the minority class often corresponds to the class of interest—for example, in toxicology prediction^45^ a metric favoring the majority class can be problematic. To counteract this behavior, balanced accuracy was introduced. It calculates the accuracy for both classes separately and returns the average, thus favoring no class.

5 $$Accuracy = \frac{TP + TN}{{TP + FP + TN + FN}}$$

6 $$Balanced\;Accuracy = \frac{{\frac{TP}{{TP + FN}} + \frac{TN}{{TN + FP}}}}{2}$$

Both metrics range from 0 to 1, 1 corresponding to perfect classification and 0 to perfect misclassification.

The F1-score denotes the harmonic mean of precision and sensitivity, also called recall^46^. As the harmonic mean is dominated by the minimum of its arguments it is both suitable as a means to calculate an average of ratios and in particular, to be used in computing a performance metric because it produces no over-optimistic evaluation of the classifier based on the maximal argument^47^.

It is defined as:

7 $$H = \frac{n}{{\mathop \sum \nolimits_{i = 1}^{n} \frac{1}{{x_{i} }}}}$$

Precision indicates the ratio between true positives and the total number of data instances that were predicted as positives:

8 $$Precision = \frac{TP}{{TP + FP}}$$

The F1-score is than calculated as follows:

9 $$F1 = \frac{2*P*R}{{P + R}},$$

where P is the precision and R corresponds to recall/sensitivity.

As can be seen from the Eqs. (3), (8) and (9) the F1-score does not account for true negative predictions, meaning that this metric can only be used to measure a model's predictive power for the positive class. Nevertheless, if the positive class is the focus of a study and/or additional metrics are employed the F1-score is a sensible choice. It ranges from 0 to 1, where the latter is achieved if all samples were correctly predicted. It results in 0 if all positive samples were misclassified^46^.

The Matthews correlation coefficient (MCC) is defined as:

10 $$MCC = \frac{TP*TN - FP*FN}{{\sqrt {\left( {TP + FP} \right)\left( {TP + FN} \right)\left( {TN + FP} \right)\left( {TN + FN} \right)} }}$$

As it is a binary classification metric that results in a high value only if the majority of both positive and negative samples are predicted correctly, it is well suited to evaluate classifiers that are either designed to achieve good predictive power for both classes or are based on an imbalanced training set. At the same time, in case that all entries of a column or row of the confusion matrix are equal to 0 the MCC is undefined^46^.

The MCC ranges from − 1 to 1, the first corresponding to perfect misclassification and the latter to perfect classification.

Cohen's Kappa was originally introduced as a measure of agreement between raters corrected by chance agreement. If used as a performance measure of binary classification, the agreement between the actual labels and the classes predicted by the classifier is measured instead^48^. It is defined as:

11 $$K = \frac{{Acc - P_{e} }}{{1 - P_{E} }},$$

where Acc is the accuracy of the classifier and P_e_ is the probability of chance agreement.

P_e_ is given by:

12 $$P_{e} = \frac{{\left( {TP + FN} \right)\left( {TP + FP} \right) + \left( {FP + TN} \right)\left( {FN + TN} \right)}}{{n^{2} }},$$

where TP + FN equals the number of positive samples, TP + FP is the number of positive predictions, FP + TN corresponds to the number of negative samples, FN + TN is the number of samples predicted as negative and n is the total number of samples.

As the definition of chance agreement in its formula has been criticized—alternative definitions have been introduced—and problematic scenarios have been identified of which most are associated with an imbalanced dataset—worse classifiers can achieve better results—Cohen's Kappa can be a somewhat unreliable metric^48^. Nevertheless, when used in combination with other metrics, especially the MCC, Cohen's Kappa can still be a valuable measure of agreement between ground-truth and assigned classes. Additionally, it is a widely employed metric and can therefore be used to compare results of different studies^48^.

Another widely established performance metric is the receiver operator characteristic area under the curve (ROC-AUC), which is based on the ROC curve. To plot this curve, first the predictions made by the classifier under consideration are sorted by their prediction probabilities for the positive class. While iterating through them the decision threshold—which would normally be set at 0.5—is changed to the respective prediction probability, in this fashion every instance is “assigned” to the BBB positive class. A point corresponding to a prediction is plotted in the graph—of which the x-axis represents the false positives rate and the y-axis the true positives rate—such that either its x or y coordinate is increased compared to the previous point in the graph by the fraction of the positive or negative class it represents (1/# positive samples or 1/# negative samples). As the first predictions that are plotted are those having the highest probabilities of belonging to the positive class, i.e. the classifier is most sure that the respective samples are positive, a well performing model would have a steeply rising curve in the beginning, indicating a high true positive rate. And since the probabilities are decreasing with each sample, meaning the proportion of negative samples increases, the curve would quickly flatten at the end, indicating a high false positive rate^49^.

The area under the curve (AUC) is a way to reduce the ROC curve to a single value, enabling easier comparison of different classifiers via this metric. Its value range is between 0 and 1, where random guessing would result in a value of 0.5, 1 indicating perfect prediction of positive samples while a value of 0 corresponds to the case that all samples are assigned to the wrong class^49^.

## Results

**Figure 5:**
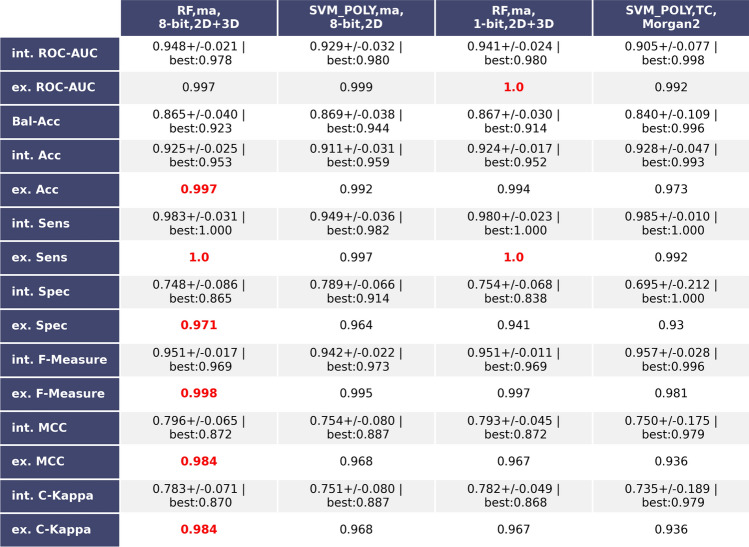
Table showing the three best performing models based on the imbalanced dataset and MI-DSE fingerprint and the best performing model based on the imbalanced dataset and Morgan 2 circular fingerprints. Both internal cross-validation (average with standard deviation) and external validation results are depicted. The highest values of each performance metric are marked in red.

Figure 5 displays the best performing models trained and internally validated on the imbalanced dataset. Of these three were based on the MI-DSE fingerprints and one on the Morgan 2 fingerprints. The Random Forest employing MI-DSE fingerprints encoding 49 2D and 3D descriptors in 8-bits each, and 11 structural keys and being trained on a dataset produced by the Mahalanobis distance based Kennard-Stone algorithm—achieved external validation scores close to 1 for all performance metrics used in this study. It is constructed using 100 decision trees with a depth of 400, while the Gini-Index was chosen to evaluate splits. It was trained on 1108 active and 366 inactive compounds, while the test set contained 325 BBB-permeating and 34 BBB-non-permeating molecules.The model is now named as bbbPythoN-imb. The exact compositions of all training and test set pairs can be found in Table 2. Features encoded in the best-performing MI-DSE fingerprint are shown in Tables S10 and S11 in the supplementary material.

Tables showing the performance metrics of all 60 models created on the imbalanced dataset can be found in the supplementary material (Tables S1–S6).

**Figure 6:**
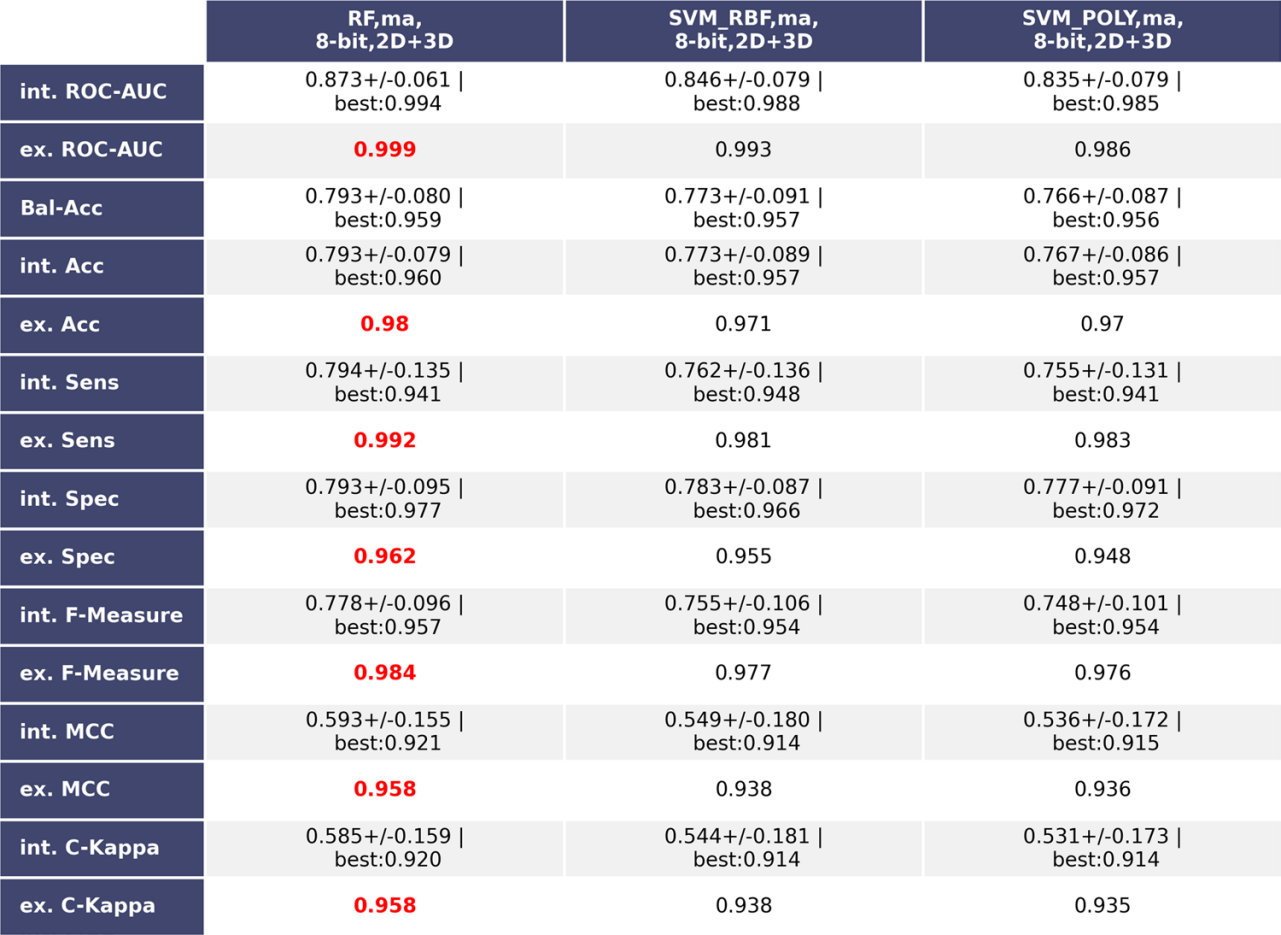
Table Showing the three best performing models based on the balanced dataset and MI-DSE fingerprints. Both internal cross-validation (average with standard deviation) and external validation results are depicted.The highest values of each performance metric are marked in red.

Depicted in Fig. 6 are the performance metrics of the three best models trained and validated on the balanced dataset composed of molecules of the B3DB dataset. Again, the RF based on 2D + 3D MI-DSE fingerprints using 8 bits to encode descriptors and trained and tested on a dataset split via KS employing the Mahalanobis distance outperformed the other models while reaching values close to one for all metrics in the external validation. This model is referred as bbbPythoN-bal from this point. However, the average internal validation results are noticeably smaller, and their standard deviations are larger than those of the bbbPythoN-imb, with the exception of the specificity. Also, the best bbbPythoN-bal of the k-Fold cross-validation outperforms its imbalanced counterpart in most cases.

Both our best models show that they are reliable predictors, even in the case of the query molecule being an outlier concerning the chemical space known to the model (see Figs. S7, and S8, and Tables S10 and S11 of the supplementary material). Overall, it can be established that our models outperformed classifiers introduced in comparable publications both in indirect (see Table 2) and direct comparisons (Fig. S7 of the supplementary material).

Finally, bbbPythoN-imb and bbbPythoN-bal were additionally externally validated on the dataset composed from the works of Li et al.^20^, Wang et al.^15^, and Tong et al.^28^ (see Fig. S8 of the supplementary material). Both models performed well. bbbPythoN-imb scores higher on sensitivity (0.963)—likely due to the active class being a strong majority in its respective dataset—while achieving a lower specificity (0.81) than bbbPythoN-bal. The latter reaches a sensitivity of 0.86 and a specificity of 0.94. Both models will be made available via our package bbbPythoN on GitHub.

### Features space analysis for CNS drugs vs non-CNS drugs

**Figure 7:**
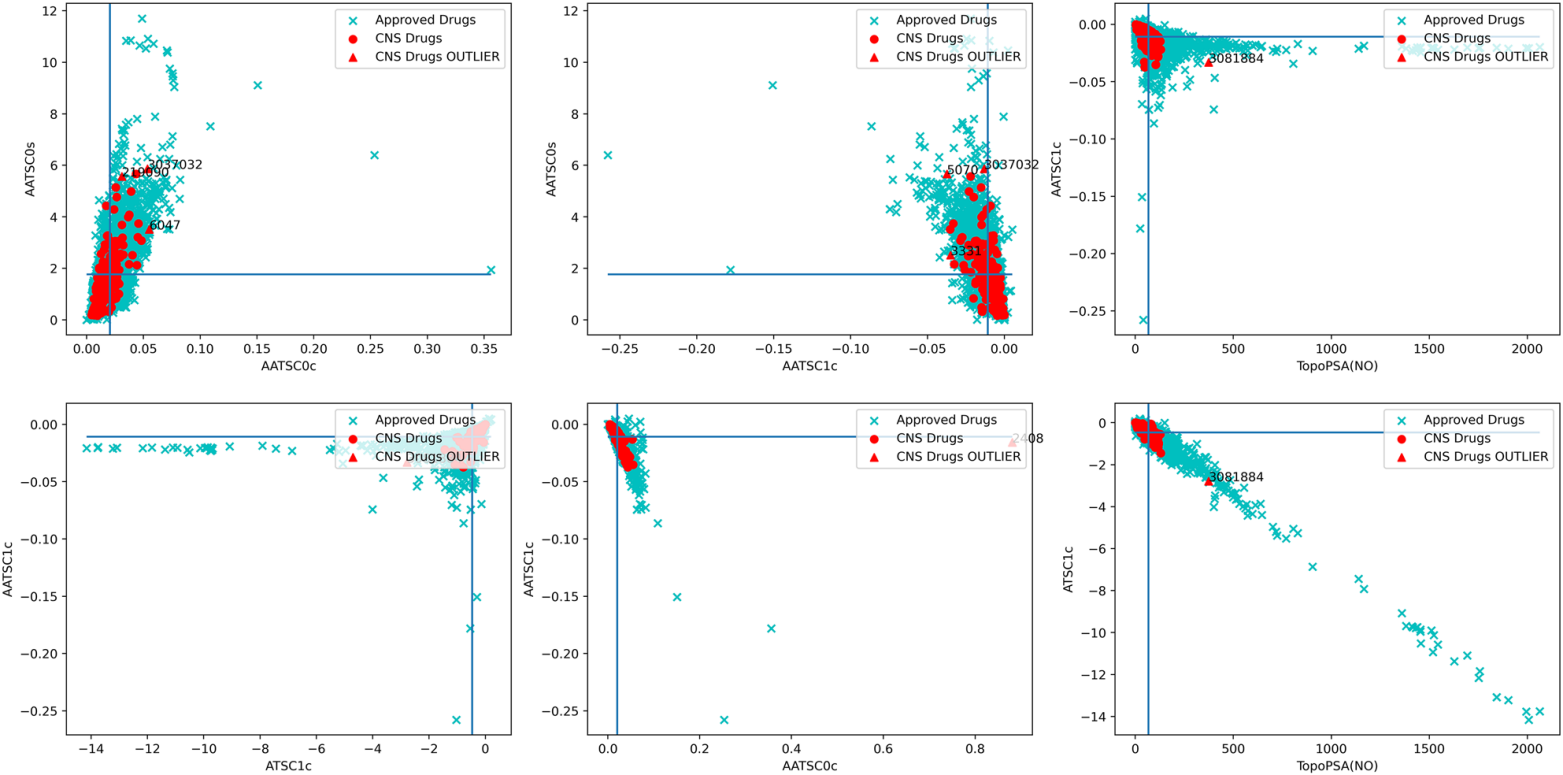
2D descriptors: Scatterplots of approved drugs and CNS drugs via six combinations of best performing 2D descriptors, AATSC0s, AATSC0c, AATSC1c, TopoPSA(NO) and ATSC1c. Stark conservation of CNS drugs around values indicating low polarity can be observed.

**Figure 8:**
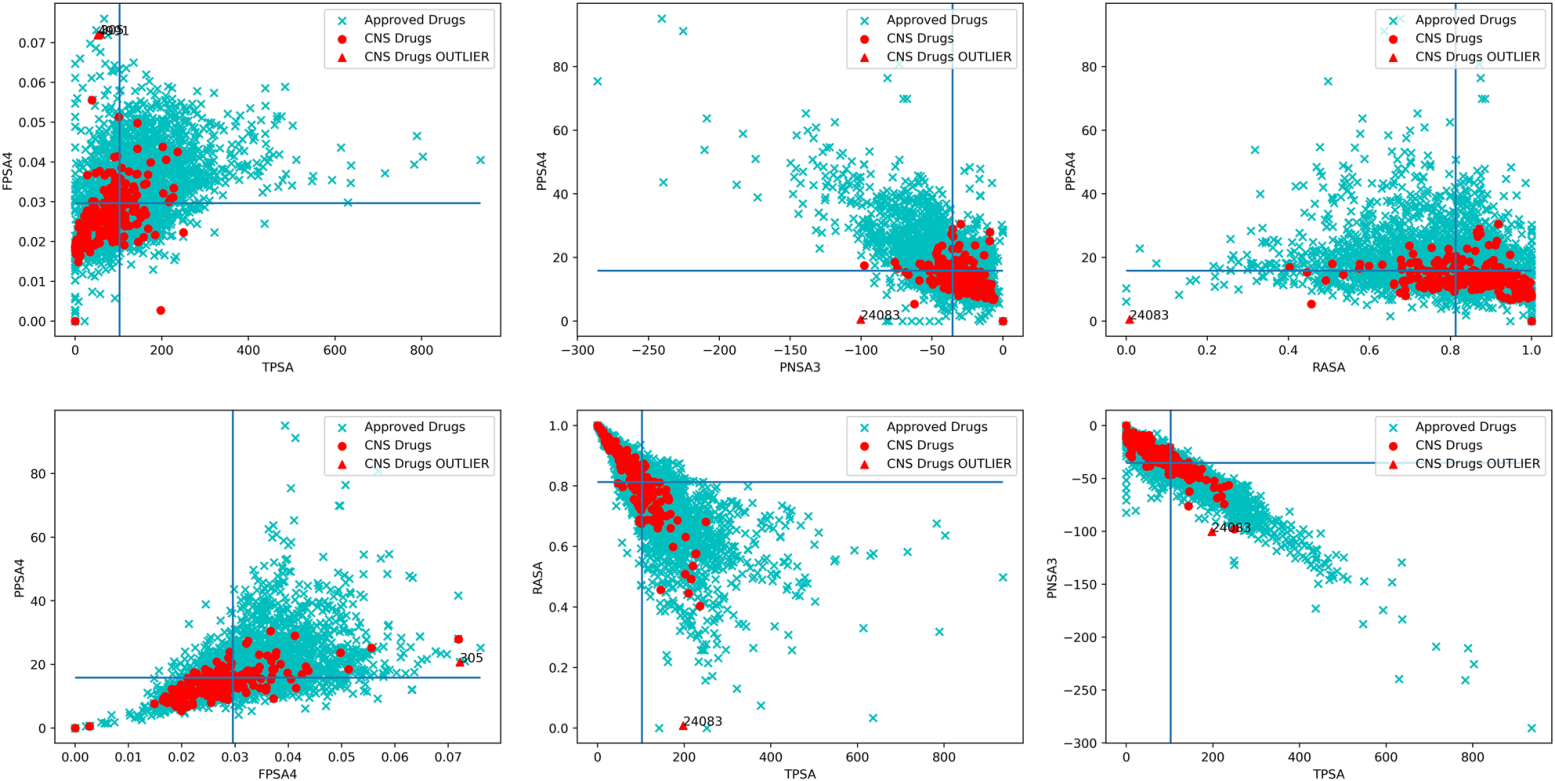
3D descriptor: Scatterplots of approved drugs and CNS drugs via six combinations of best performing 3D descriptors, FPSA4, TPSA, PPSA4, PNSA3 and RASA. When compared with Fig. 5(2D) the CNS drugs are less conserved but tend to lower values for TPSA, FPSA4 and PPSA4—all relating to charged surface area—and higher values for RASA being a measure of hydrophobic surface area.

Initial analysis of CNS drugs vs non-CNS drugs using simple physicochemical descriptors revealed differences in the descriptor spaces. The initial physicochemical properties-based analysis is independent of molecular conformation and therefore may not account for many important features like internal hydrogen bonding etc. Additionally, to understand how well the top-performing features selected in the current method can distinguish the CNS from the non-CNS drugs, we repeated the same analysis using those 2D and 3D descriptors that were found to discriminate best between classes. As shown in Figs. 7 and 8, CNS drugs (red) and approved (non-CNS) drugs (blue) are well differentiated using the selected descriptors. 2D descriptors like AATSC1c, ATSC1c, AATSCOc and TopoPSA(NO) represent the CNS space drugs well. Similarly, 3D descriptors—PPSA4, PNSA3, RASA and TPSA are more conserved for CNS drugs. A similar analysis was performed for the CNS active, and CNS inactive compounds present in the training set used in this study and information obtained was used in training the models.

## Discussion

In this study, multiple subsets of 2D and 3D descriptors calculated via the Mordred descriptor calculator^36^ were chosen employing the MI-DSE feature scoring and encoded as property-based fingerprints to predict BBB-permeability of molecules. For comparison, Morgan circular fingerprints with a radius of 2 were applied to the same task.

Two base classifiers (SVM and RF) were used to build models, each subjected to 10-fold cross-validation and external validation. Due to the use of two datasets, application of multiple fingerprints—employing different descriptors and numbers of bits to encode their values—Kennard-Stone dataset splitting based on either Euclidean or Mahalanobis distance, and finally SVMs with both RBF and Polynomial kernel, 63 distinct models were trained. We have compared our best performing models to other published models shown in Table 2. The respective published model’s accuracy, sensitivity, specificity, AUC-ROC and MCC if supplied by the respective authors, are also reported. These metrics were chosen for their ability to represent the relevant properties of a binary classifier and their widespread use in gauging classification results. A detailed description of these is given above under Model Performance. Though the top 3 models achieved relatively similar results, our models outperformed the other classifiers.

It can be seen that all publications, including our own, employed imbalanced datasets in which the actives represented the majority class (see Table 1). This is mirrored by the classifiers having higher sensitivities than specificities, with the exception of Alsenan et al.’s model^13^, due to bias introduced by data imbalance. Alsenan et al.^13^, Liu et al.^19^, and Wang et al.^15^ tried to negate this effect via SMOTE (Synthetic Minority Over-Sampling Technique) oversampling of the minority class, which only in the case of the former alleviated the problem. It even led to their model’s specificity outscoring its sensitivity. Nevertheless, synthetic samples produced by SMOTE are created by randomly choosing existing minority samples, finding their k-nearest neighbours and setting the synthetic feature values to the most common value. Thereby the feature space of the minority class is reproduced and possibly even further conserved, risking both overfitting and overestimation of the classifier’s performance. At the same time, an imbalanced dataset also poses the risk of overestimation due to the minority class having less impact on the performance metrics. This is why we opted to collate an additional balanced dataset from the B3DB dataset^27^.

The effects of imbalanced data in general and in regard to our classifiers are further discussed below. To investigate the quality of the feature selection set and whether and how MI-DSE scores are affected by its composition, multiple feature selection sets of varying ratios between BBB actives and BBB inactives were produced. The classes were either kept in their original form, extended via samples taken from the supplementary material of Wang et al.^15^ in case of the BBB negative class, or down sampled by randomly choosing a subset in case of the BBB positive class. The effect of the different compositions was measured by calculating the MI-DSE score of the TopoPSA(NO) descriptor.

The TopoPSA(NO) descriptor—the topological polar surface area only considering oxygen and nitrogen atoms—was chosen as it achieved the highest MI-DSE score of all features. This is in concordance with multiple publications^11,12,14,50^ describing it as well suited to distinguish between bb-barrier permeating and non-permeating compounds.

**Figure 9:**
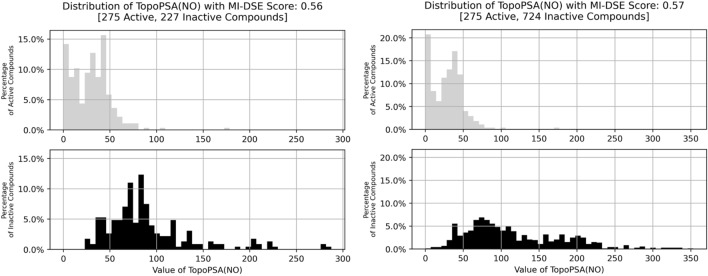
Histograms showing value distributions of TopoPSA(NO) descriptor for the positive and negative class and the respective MI-DSE scores. Left: Distributions for the original feature selection set. Right: distributions for dataset with an extended negative class.

A larger negative class would, due to its non-specificity in regard to activity, encompass an increase in its chemical diversity, which we suspected would also lead to a significant improvement of MI-DSE scores or change of high-scoring descriptors. From Fig. 9 it can be observed that, though the value distribution of TopoPSA(NO) for the negative class is much more evenly spread across the corresponding value range, the MI-DSE did not increase significantly. In addition, we observed that the reduction of positive samples led to a stark decrease of the MI-DSE score, which dropped by almost half to 0.3.

Overall, it seems that the employed feature selection set was well suited for the given task and could not have been significantly improved with the data available.

A classifier's predictive power and applicability domain (AD) are always limited by the size of and chemical space projected by the training dataset. Meaning the larger the chemical space covered by the dataset the greater the AD of a model. In case new compounds are not part of a model’s AD, predictions of these samples should be assumed unreliable. To be able to assess the reliability of such predictions, a measure of similarity between a newly predicted compound and the training dataset should be supplied as well as the respective prediction probability. Since the Mahalanobis distance has been proven to be a metric well suited for outlier detection^39^ it will be supplied for predictions of new compounds made by classifiers created in the process of this work.

As the training dataset of the bbbPythoN-imb classifier is imbalanced, the active compounds being the majority class, it is probable that the dataset is less chemically diverse than a balanced dataset of the same size, since the active class can be expected to be more chemically conserved, in turn reducing the extent of the AD in regard to inactive compounds of the resulting models. Nevertheless, because the bbbPythoN-imb model was designed to be focused on identifying molecules able to penetrate the bb-barrier and the specificities of the models were not substantially suffering in internal and external validation, the positive compounds representing the majority class do not seem to influence model performances strongly. However, when comparing the performances of bbbPythoN-imb and bbbPythoN-bal on the dataset compiled from Li et al.^20^, Wang et al.^15^, and Tong et al.^28^ the above effect can be observed for the bbbPythoN-imb model. Simultaneously the balanced dataset had a strong positive effect on predictions of the negative class of the bbbPythoN-bal classifier. In contrast, its ability to detect positive class samples suffered compared to its sensitivity on the balanced B3DB^20^ dataset. This nicely shows the impact of the dataset compositions on a model’s AD and how it can lead to a classifier developing a focus for one of the classes present in the data. The comparison also shows that even if a balanced dataset is used it is no guarantee for the model not to develop a bias. Overall, to increase a model’s AD, it is sensible to collate a dataset consisting of chemically diverse molecules that is reasonably balanced and large. Additionally, sampling can be employed in case of imbalanced datasets to reduce the risk of model bias towards the majority class^45^. Moreover, can a model’s AD be evaluated by predicting compounds of external test sets, assessing the quality of the predictions—their correctness and respective prediction probabilities—and comparing the chemical spaces of both the training and external datasets.

As can be seen in Fig. 5 the imbalanced dataset led to the bbbPythoN-imb model developing a bias towards the majority class. However, there is a stark increase in external validation specificity (Fig. 5), which could be explained by the Kennard-Stone algorithm being employed for dataset splitting. It is designed to produce training sets recreating the original dataset’s chemical space as closely as possible, the consequence of which is chemically restricted test sets which consist of more conserved and therefore, easier-to-predict compounds. The improvement of specificity between internal and external validation could additionally be caused by applying the internally best performing models to the test set. Which can be seen to clearly outperform the average internal model (Figs. 5 and 6). An overall increase in external performance metrics can also be observed for the bbbPythoN-bal model (Fig. 6), which can be explained by the same reasoning as above.

The Kennard-Stone data splitting technique also led to the test sets of this study containing smaller numbers of inactives, especially in the case of the imbalanced dataset (see Table 2). Though the activities were incorporated into the Mahalanobis distance calculation, their impact on the class distributions seems to have been marginal.

Nevertheless, the F1-scores, being the harmonic mean of precision and sensitivity, of our bbbPython-imb model reaches values around and above 0.95 in internal and external validation, respectively. Indicating a high reliability of positive predictions. It has to be mentioned, though, that due to the low number of inactives in both training and test sets the impact of specificity and FPs on the other performance metrics, including the F1-score, is low. At the same time, the high specificity for the test set validation with only 34 inactive samples shows that only one of these has been misclassified as active. In general, when dealing with a small inactive class, few FPs would already cause a strong decrease in specificity. For the bbbPythoN-bal model, the inactives were much better represented in the test set with 479 BBB-permeating and 286 BBB-non-permeating compounds. Its estimation of predictive power for the negative class can therefore be assumed as more reliable, with a specificity of 0.962.

Overall models based on 2D descriptors were found to be much more predictive of bb-barrier permeability, as can be seen from Figure S1 to S6 of the supplementary material.

When the top ten features of both the 2D and 3D descriptors are considered, one can observe that the top features of each set—TopoPSA (NO) and total polar surface area (TPSA)—are very similar in what chemical properties they represent. The 2D set is dominated by autocorrelation descriptors, representing the distributions of specific properties along the molecular (topological) structure as well as the correlation of property values of neighboring atoms (defined by the lag) in the case of the Geary coefficient, while the 3D set mostly comprises of features describing either fractional, relative or partial surface areas of positive or negative polarities weighted by differing schemes.

Since the properties measured by many of the autocorrelation descriptors in the top ten 2D features, like Gasteiger partial charge and Sanderson and Pauling electronegativity, are related to molecular polarity which in turn influences hydrophilicity, hydrophobicity and lipophilicity, and TopoPSA(NO), TPSA as well as the 3D polar surface area descriptors are also connected with these molecular properties, there is a strong indication of their relevance to bb-barrier permeability. This seems logical because of the overall importance of non-polarity for the process of passive membrane crossing of molecules.

Those autocorrelation descriptors do not employ partial charge or electronegativity directly as weights are using the intrinsic state, which also incorporates electronegativity in its formula, and the number of valence electrons, indicating reactiveness and tendency to form bonds. The remaining top ten 2D descriptors are the IC1, the 1-ordered neighbourhood information content measuring the complexity of a molecular graph, and SdO, being the sum of E-sate values of oxygen atoms having one double bond, where E-sate values describe reactiveness, interactivity and buriedness of a molecule’s atom.

The top ten 2D descriptors, in general show a more diverse depiction of physico-chemical properties, even if an overlap of these depicted properties can be noticed when compared with the set of 3D descriptors. The same is true when considering the complete 2D and 3D descriptor sets, indicated by the overall higher correlations between the 3D features. Moreover, is the set of 2D features chosen by the feature selection significantly more numerous than the set of chosen 3D descriptors (2D set: 48 descriptors, 3D set: 16 descriptors), which can be explained by the on average lower MI-DSE scores of the latter leading to a higher number of rejected descriptors due to the used threshold. Therefore, the 2D descriptor set relays broader and more diverse information to the classifiers.

**Figure 10:**
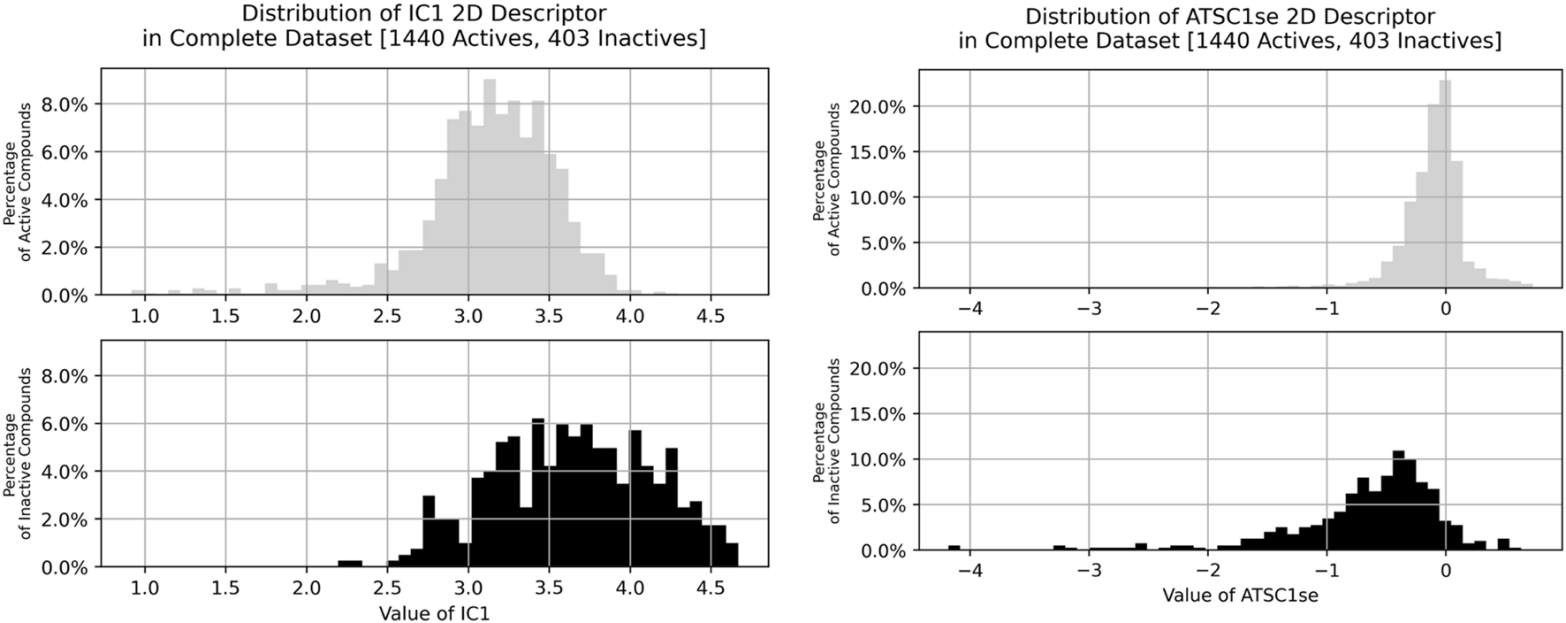
Histograms of IC1 and ATSC1se values for active and inactive classes. As described above it can be seen that the active class samples are conserved around specific values, while the inactive class is more widely distributed. This indicates the ability of these descriptors to discern between the respective activity classes while also showing that active compounds tend to be both simpler (IC1) and less polar (ATSC1se) than inactive samples.

In the plot of the value distributions of the ATSC1se descriptor, being the centered Moreau-Broto autocorrelation of lag 1 weighted by the Sanderson electronegativity, of the actives and inactives of the complete dataset (Fig. 10-left) it can be observed that the active compounds are strongly conserved around a value of 0.0.

Since only the atom pairs having a graph distance of 1 are being considered, they are bonded directly to each other. The centred values of their respective electronegativity are multiplied, i.e. their differences to the molecular mean electronegativity, a value close to 0.0 of the resulting descriptors indicates a generally low disparity between the electronegativities of the atoms leading to a low or no polarity of the overall molecule. Such low or non-existent polarity is, as stated earlier, important for a molecule’s ability to cross membranes, which is additionally underlined by the stark conservation of the active compounds seen in Fig. 10.

All the aforementioned observations suggest passive permeation as major process of bb-barrier crossing for CNS-active molecules, which is additionally supported by the statement of Pajouhesh et al.^33^ “There is a molecular recognition event when a drug approaches the external polar face of the BBB. Given the diverse nature of the CNS drugs, this interaction is more of a physiochemical nature than steric, i.e. it does not have the stricter steric requirements that a receptor-ligand pair would have”.

Since the 2D descriptors employed and encoded in the fingerprints seem to be better suited to display the properties necessary for passive crossing of the bb-barrier—as stated above—which appears to be the main process of passing the bb-barrier in the dataset used in this work, it is sensible that they would outperform their three-dimensional counterparts.

Due to the reasons given above, our models display a high discriminative power for the prediction of a molecule’s passive permeability, whereas the task of predicting receptor-mediated transport presumably lies outside its applicability domain. The Mahalanobis distance to the (active) training data—supplied for every new prediction—should help to ensure that a positive prediction does indeed pertain to passive permeation or indicate molecules which are likely to pass the BB-barrier via a different process.

## Conclusion

In this work, a large dataset and a separate feature selection dataset was used to train the models for the prediction of BBB permeability of small drug-like molecules. Numerous physicochemical descriptors were selected and statistically relevant features using correlation filter and data sampling techniques were used to select the significant molecular descriptors for effective fingerprint design.

The models were validated using tenfold cross-validation. All the best models achieved an accuracy, sensitivity, and AUC-ROC of above 95% on both cross-validation and external validation set.

The BBB model presented in this study aims to facilitate the BBB predictions in the CNS drug discovery process and will be made freely available via ProTox webserver, a computational toxicity prediction platform, as well as a Python package.

In this work, a balanced feature selection set was used to identify sets of 2D and 3D physicochemical descriptors relevant to blood–brain barrier permeation via MI-DSE feature importance. These features were further filtered employing the Pearson correlation coefficient in order to remove redundant information and encoded into fingerprints.

Based on these fingerprints, multiple classifiers were tenfold cross-validated and externally validated on both an imbalanced and a balanced dataset which were split into training and test set using the Kennard-Stone algorithm. The performances of the top performing models (bbbPythoN-imb and bbbPythoN-bal) in internal, external and additional external validation on a balanced dataset compiled from the works of Li et al.^20^, Wang et al.^15^, and Tong et al.^28^ were compared and the influence of the dataset compositions on the respective AD discussed. It was shown that bbbPythoN-imb, due to its dataset imbalance, developed a bias towards the active class. While maintaining good specificities in all three validations. The bbbPythoN-bal model also developed a bias, but to a lesser degree, towards the inactive class. Probably due to the dataset composition in regard to physico-chemical diversity.

All top-scoring models achieved an accuracy, sensitivity, and AUC-ROC of above 95% in both cross- and external validation.

Both the bbbPythoN-imb and bbbPythoN-bal models presented in this study aim to facilitate the CNS drug discovery process and will be made freely available via ProTox, a computational toxicity prediction platform, as well as a separate python package on GitHub.

### Supplementary Information

Supplementary Information 1.

Supplementary Information 2.

## Data Availability

The BBB models will be made available via ProTox-II platform upon publication (https://tox-new.charite.de/protox_II). The dataset used in this study has been provided with the supplementary information. The program is also freely available as a python package via this link GitHub—https://github.com/fdehnbostel/bbbPythoN.

## Abbreviations

AATSC1c: Averaged and centered Moreau-Broto autocorrelation of lag 1 weighted by Gasteiger charge
AATSC0c: Averaged and centered Moreau-Broto autocorrelation of lag 0 weighted by Gasteiger charge
AD: Applicability domain
ADMET: Adsorption, distribution, metabolism, excretion and toxicity
ATSC1c: Centered Moreau-Broto autocorrelation of lag 1 weighted by Gasteiger charge
ATSC1se: The centered Moreau-Broto autocorrelation, with lag 1 and weighted by Sanderson electronegativity
BBB: Blood brain barrier
CNS: Central nervous system
ED: Euclidean distance
E-state: Electrotopological state
FDA: U.S. Food and Drug Administration
FN: False negatives
FP: False positives
HBA: Hydrogen-bond acceptors
HBD: Hydrogen-bond donors
IC1: 1-Ordered neighborhood information content
KNN: K-nearest neighbors
KNIME: Konstanz Information Miner
KS: Kennard-Stone
log BB: Logarithm of the brain-to-blood drug concentration ratio at the steady state
log P: Logarithm of the partition coefficient
MACCS: Molecular Access System
MINtsC: Minimal E-state value of carbon atoms with one triple and one single bond
MAXtsC: Maximal E-state value of carbon atoms with one triple and one single bond
MCC: Matthews correlation coefficient
MD: Mahalanobis distance
MDEO-11: Molecular distance edge descriptor between primary oxygen atoms
MI-DSE: Mutual Information Differential Shannon Entropy
MINtsC: Minimal E-state value of carbon atoms with one triple and one single bond
NaN: Not a number
PCC: Pearson correlation coefficient
PNSA3: Partial negative surface area
PPSA4: Partial positive surface area
PubMed: Public MEDLINE
RASA: Relative hydrophobic surface area
RBF: Radial basis function
RF: Random forest
ROC-AUC: Receiver operator characteristic area under the curve
SdO: Sum of E-states of oxygen atoms with one double bond
SE: Shannon entropy
SIDER: SIDe effect resource
SMILES: Simplified molecular input line entry specification
SMOTE: Synthetic minority over-sampling technique
SVM: Support vector machines
TC: Tanimoto coefficient
TN: True negatives
TopoPSA(NO): Topological polar surface area only considering nitrogen and oxygen atoms
TPSA: Topological polar surface area
TP: True positives
