# Supplementary material for: Non-animal models for blood–brain barrier permeability evaluation of drug-like compounds
Source: Sci Rep. 2024 Apr 17;14:8908. doi: 10.1038/s41598-024-59734-9 (PMC11024088; doi:10.1038/s41598-024-59734-9)
Supplement: Supplementary file 2 — Supplementary Information 2. [file 41598_2024_59734_MOESM2_ESM.docx]

***Supplementary Information***

Non-Animal Models for Blood-Brain Barrier Permeability Evaluation of Drug-Like Compounds

Frederic O. Dehnbostel^1^, Vaibhav A. Dixit^2^, Robert Preissner^1^ and Priyanka Banerjee^1^*

^1^ Institute for Physiology, Charité – University Medicine Berlin, Berlin, 10115, Germany

^2.^ Department of Medicinal Chemistry, National Institute of Pharmaceutical Education and Research, Guwahati, (NIPER Gu-wahati), Department of Pharmaceuticals, Ministry of Chemicals & Fertilizers, Govt. of India, Sila Katamur (Halugurisuk), P.O.: Changsari, Dist: Kamrup, Pin: 781101, Guwahati, Assam, India

**Table S1: Results of Random Forest (RF) and Euclidean distance**

**
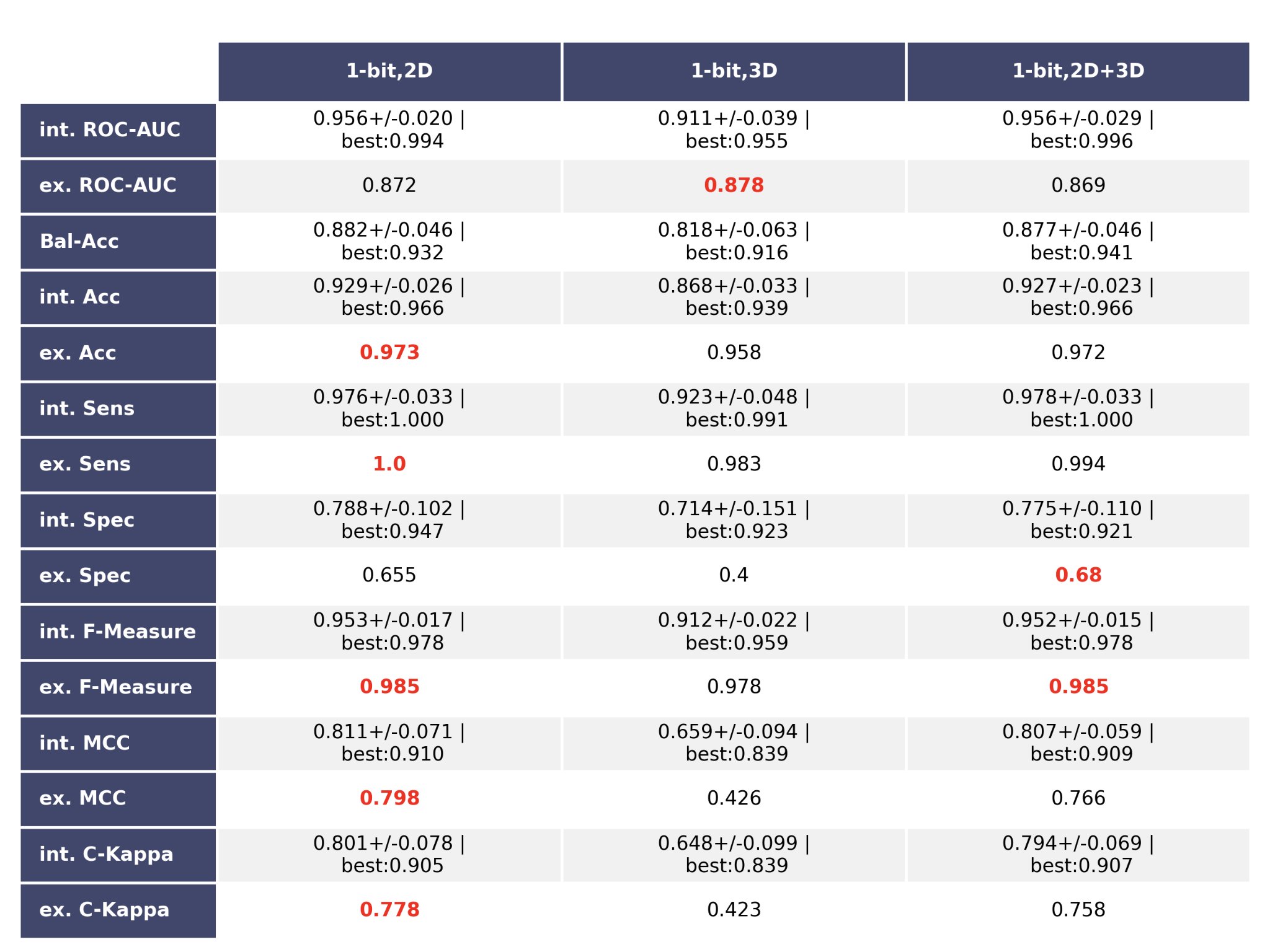
**

**
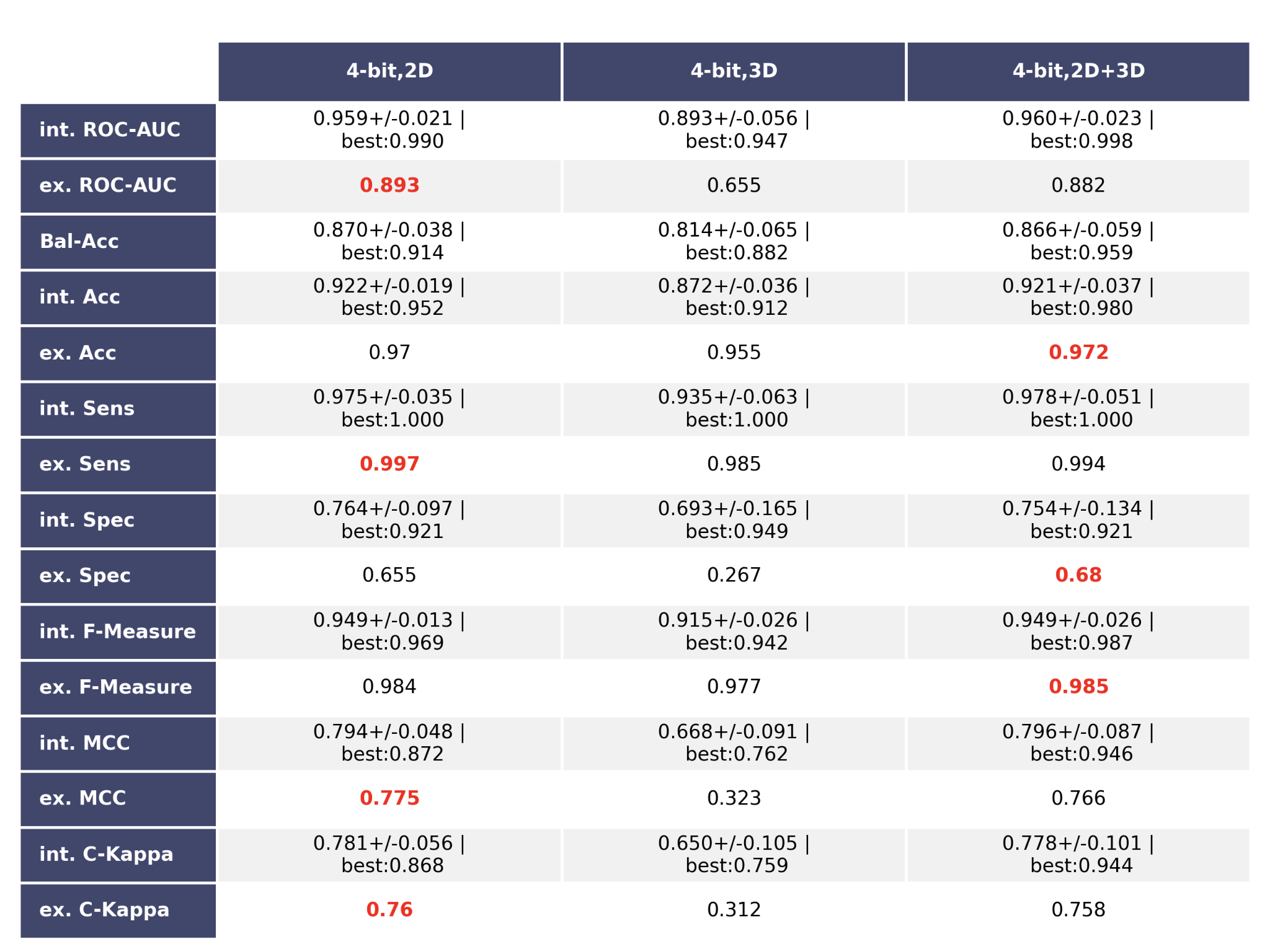
**

**
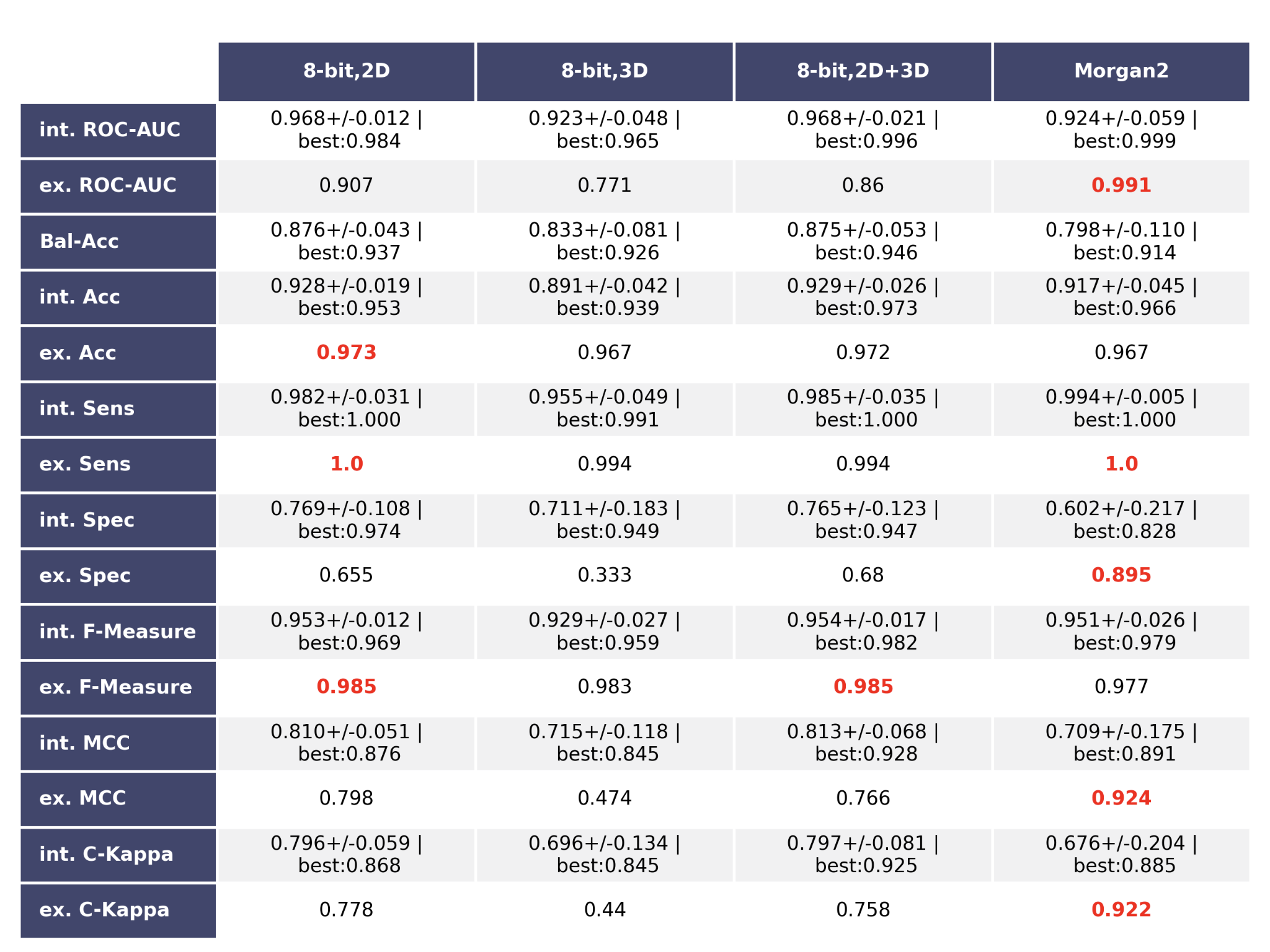
**

**Table S2: Results of Polynomial Kernel Support Vector Machine (SVM) and Euclidean distance**

**
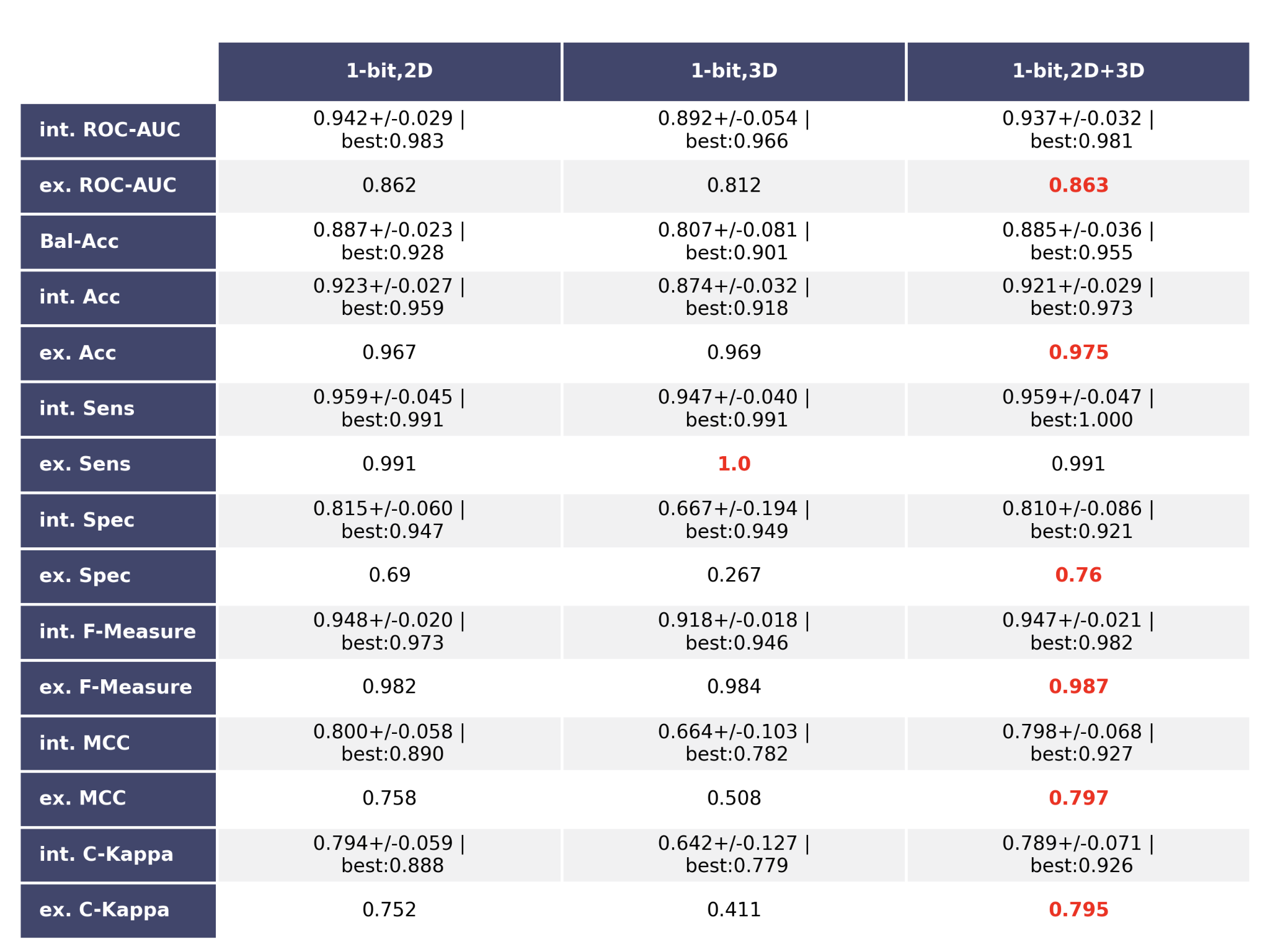
**

**
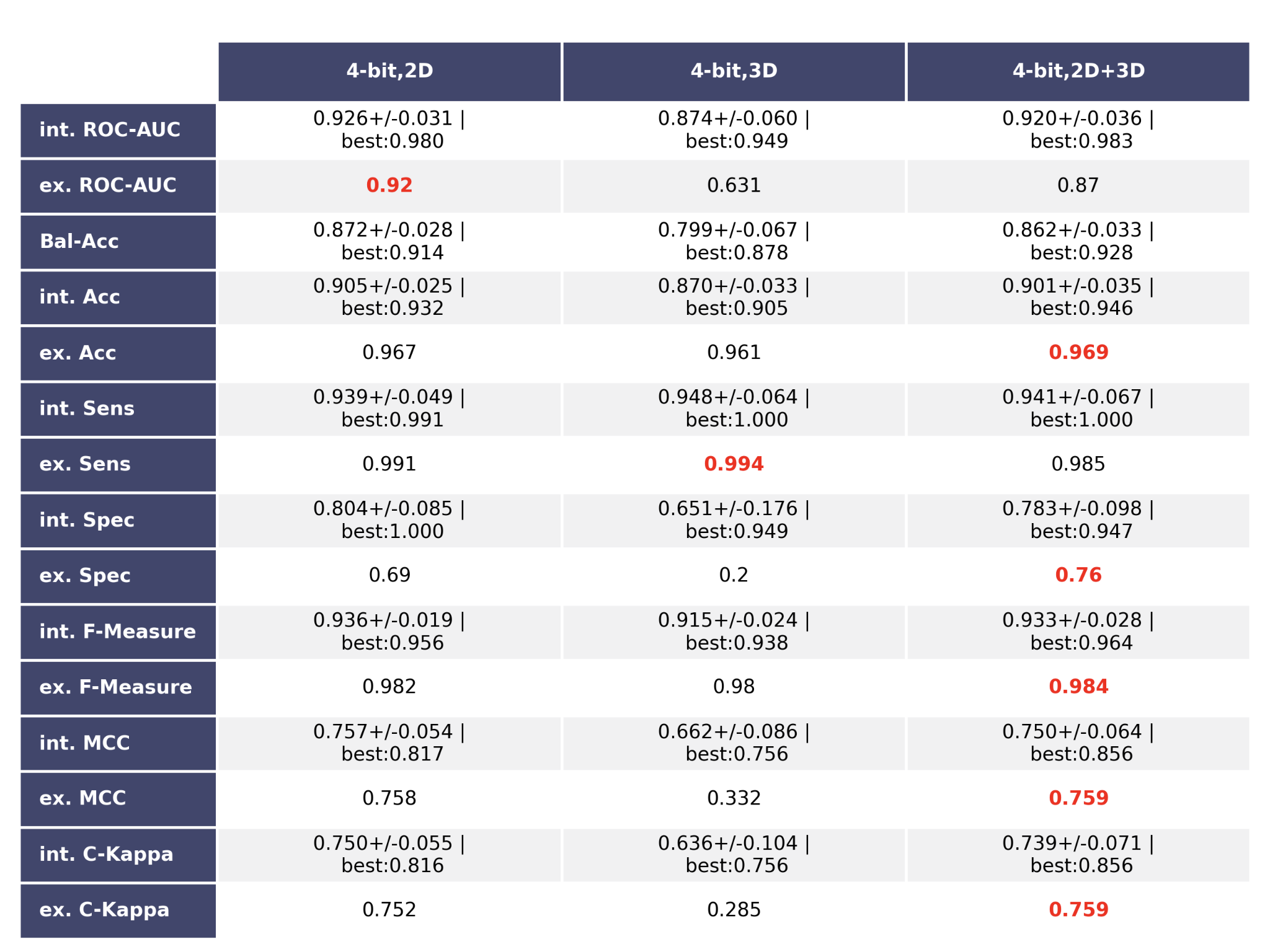
**

**
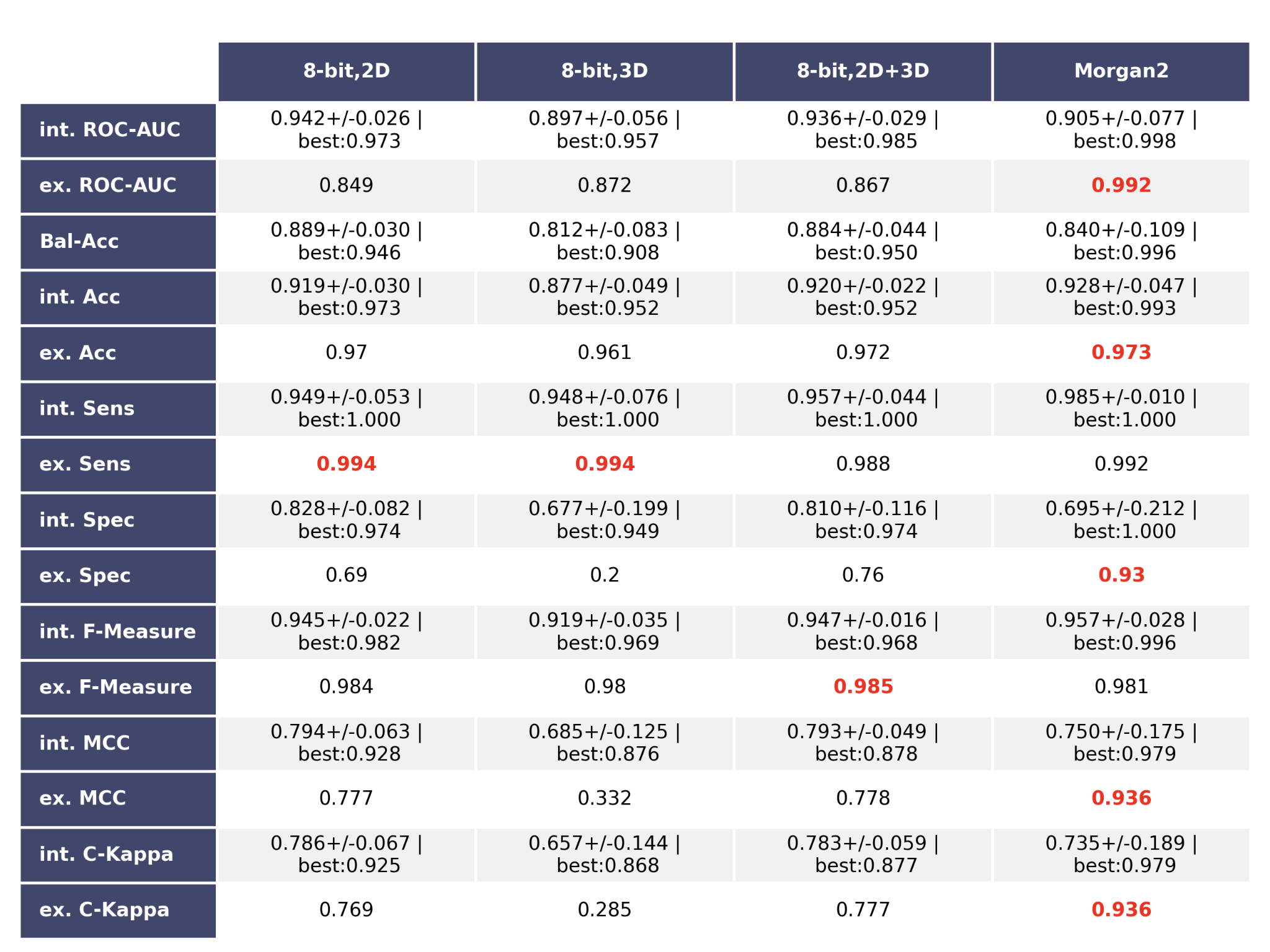
**

**Table S3: Results of Radial Basis Function (RBF) SVM and Euclidean distance**

**
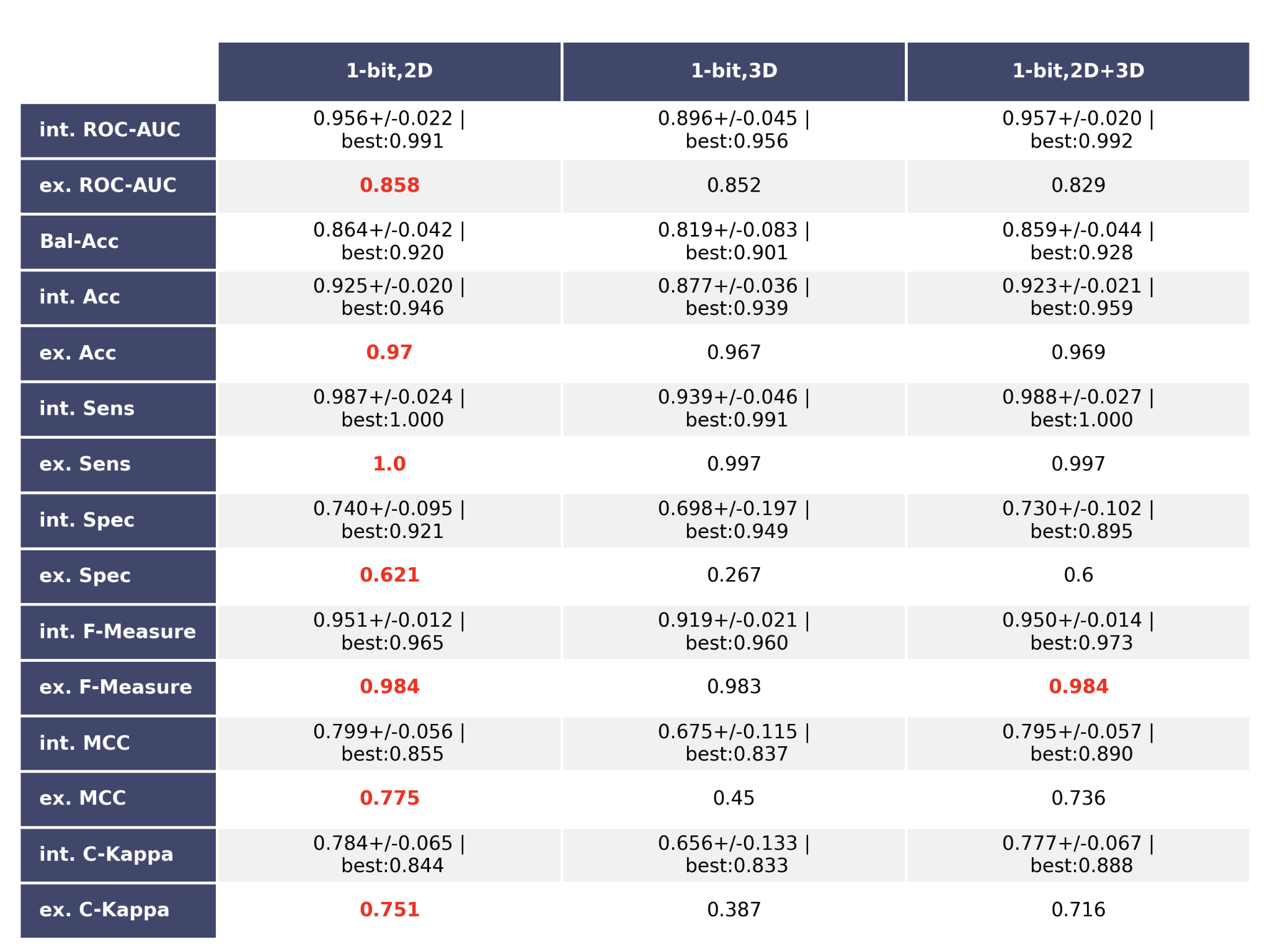
**

**
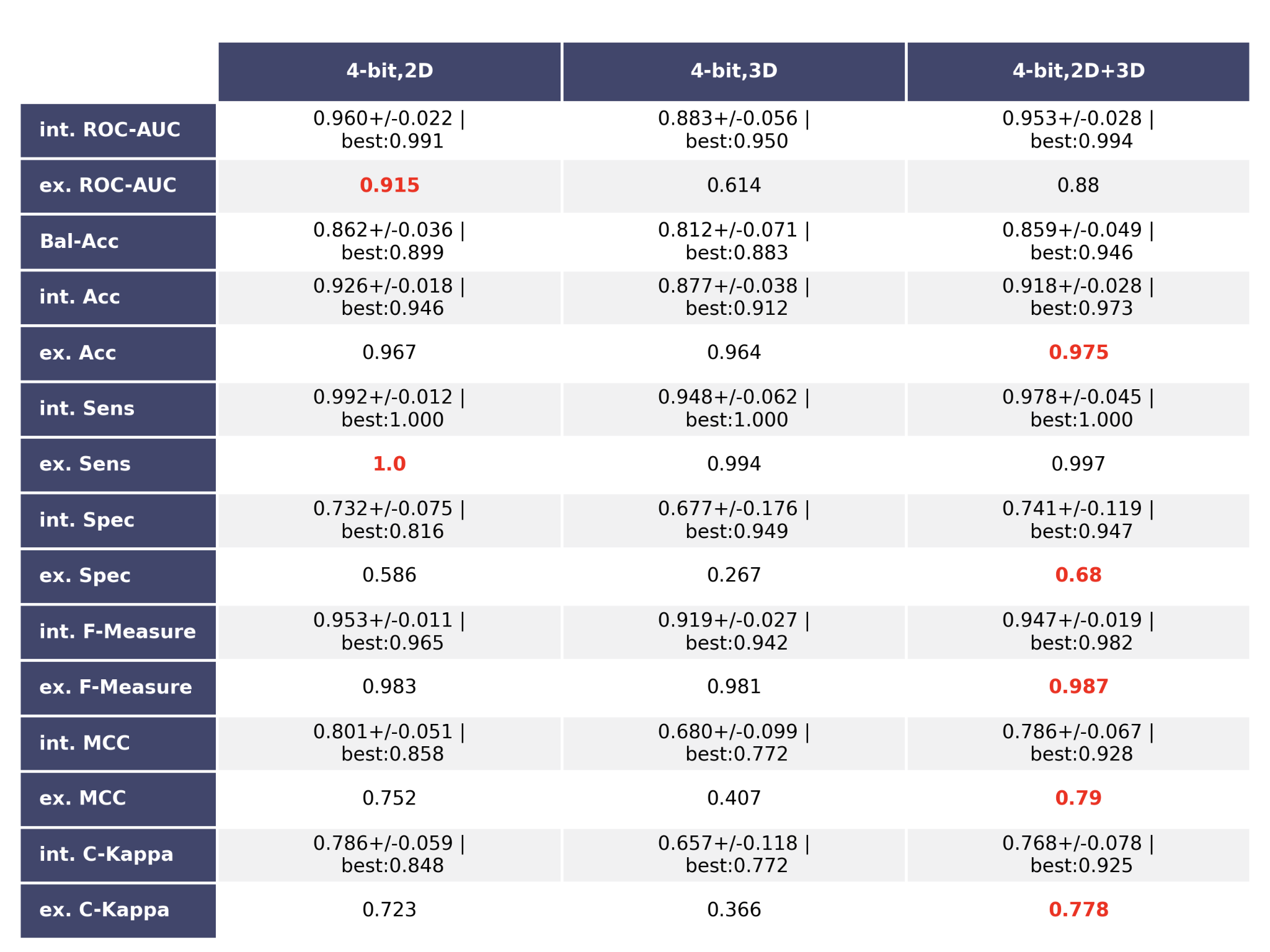
**

**
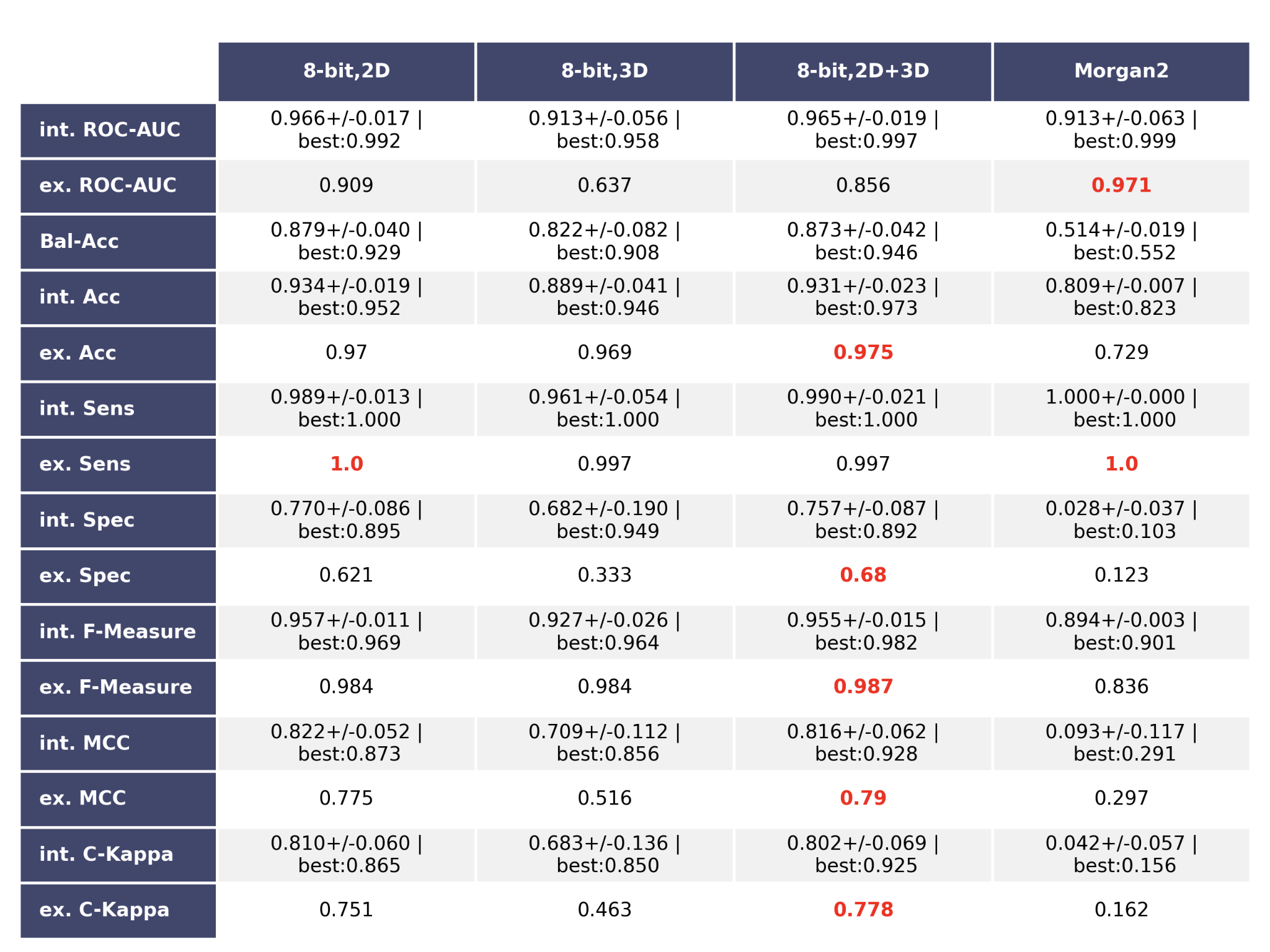
**

**Table S4: Results of RF and Mahanalobis distance**

**
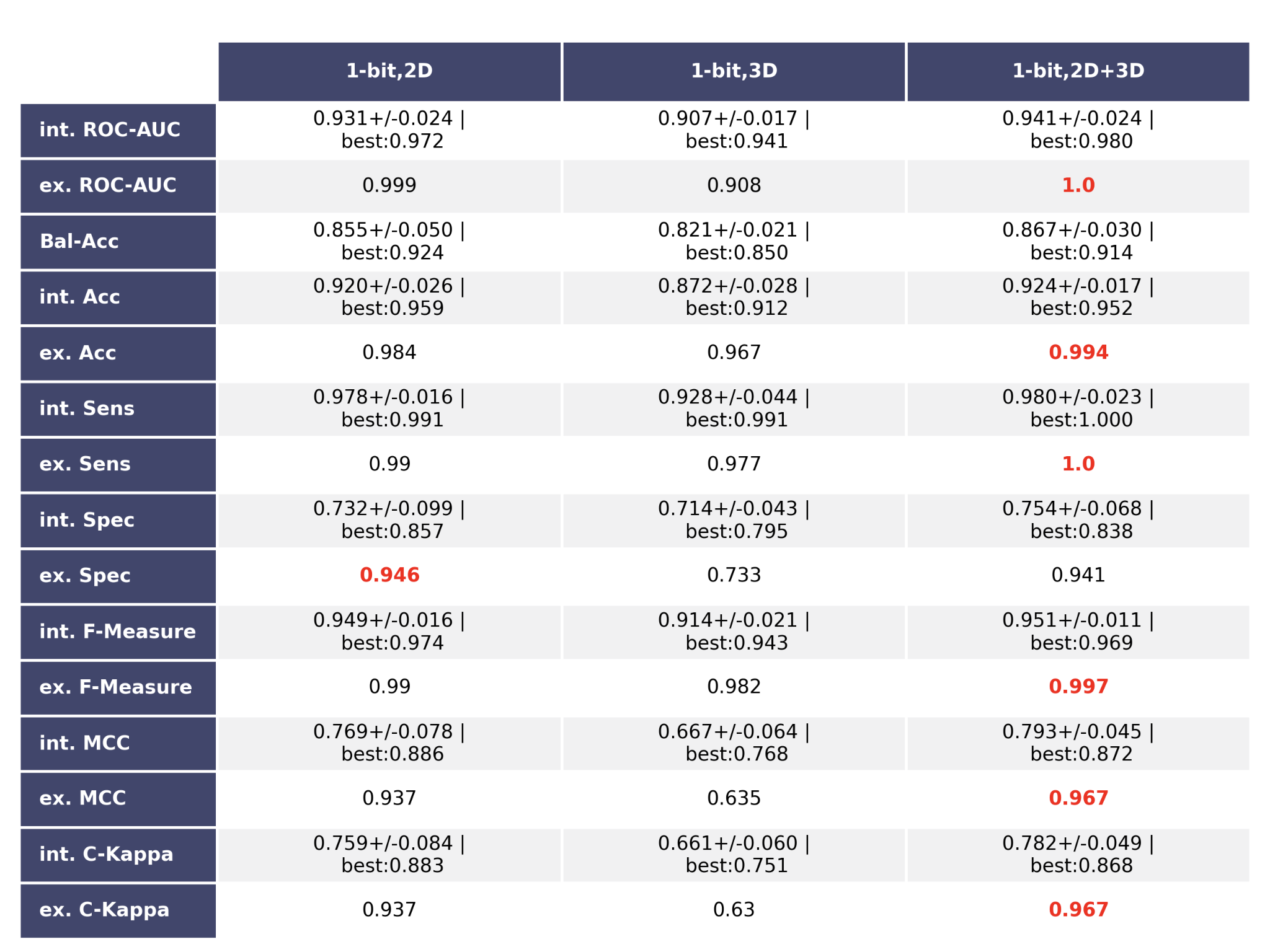
**

**
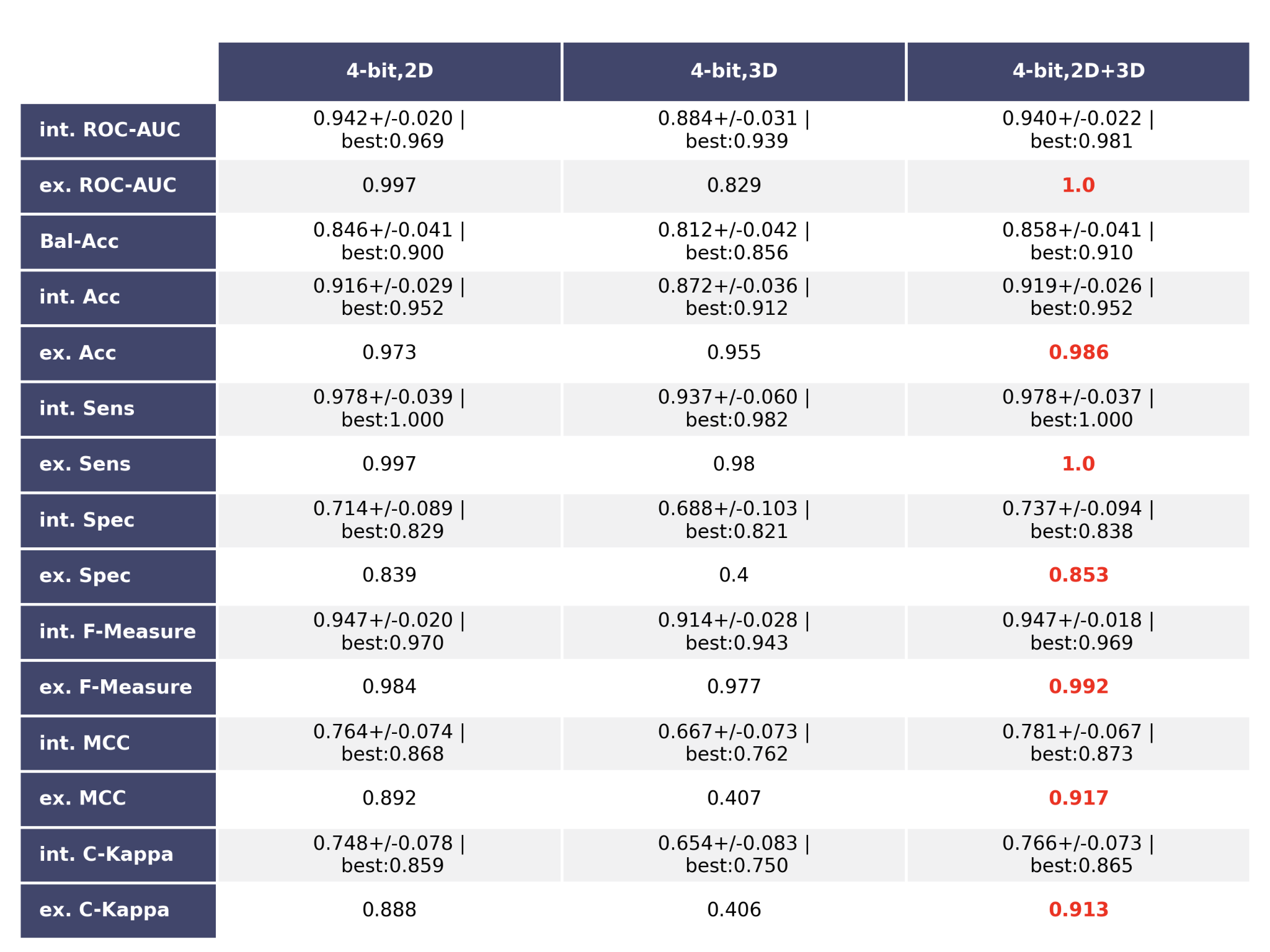
**

**
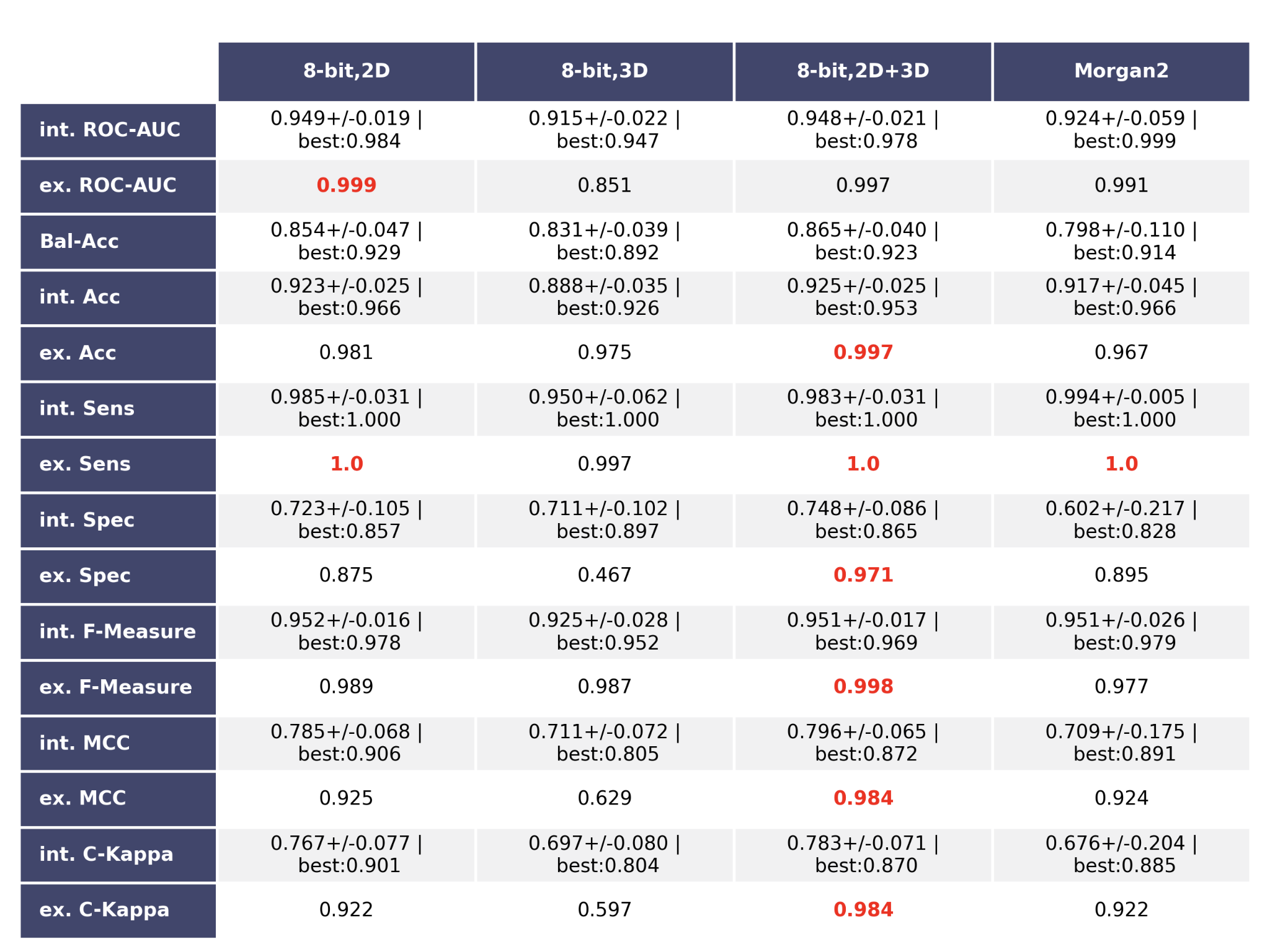
**

**Table S5: Results of Polynomial Kernel SVM and Mahanalobis distance**

**
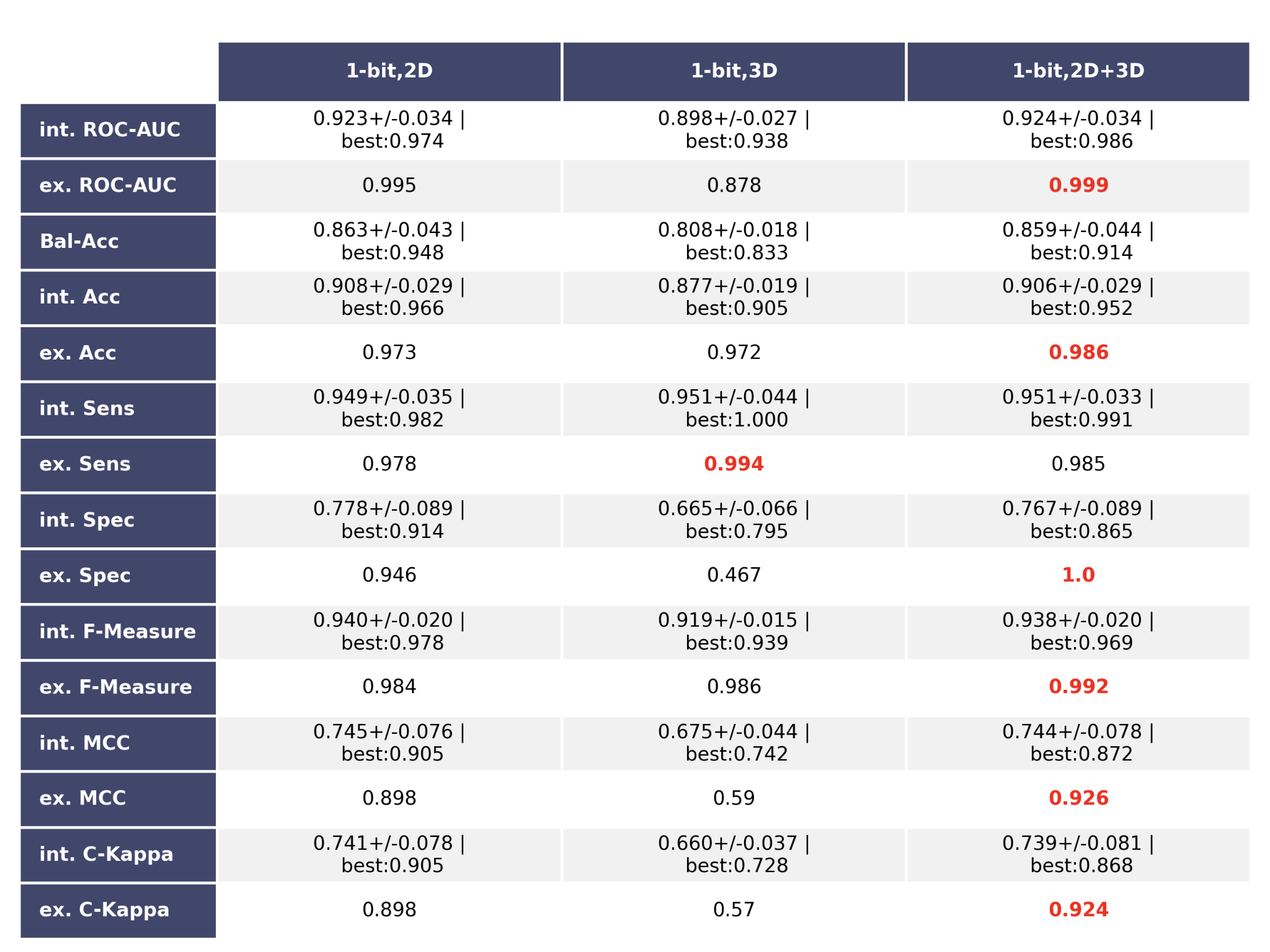
**

**
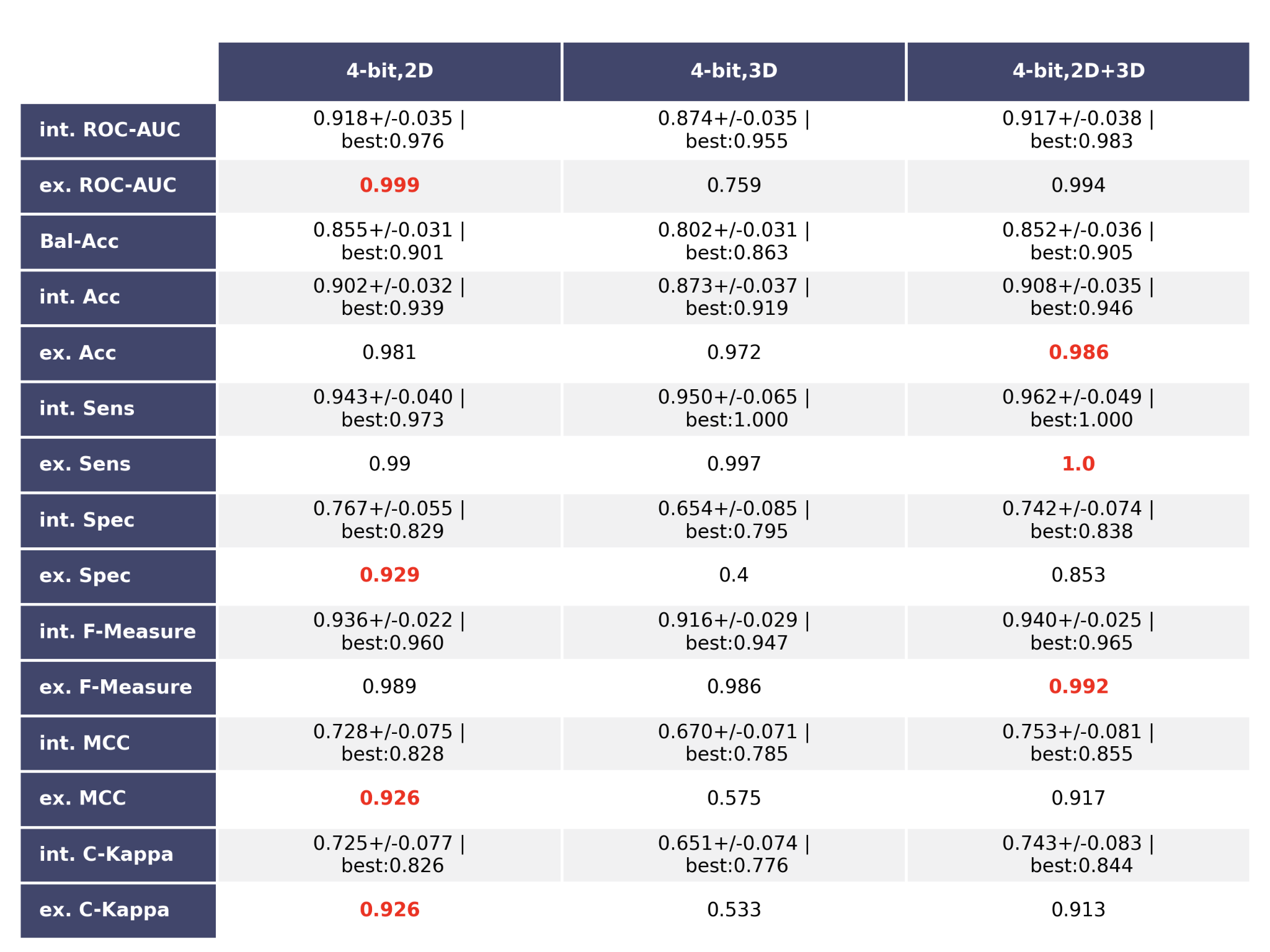
**

**
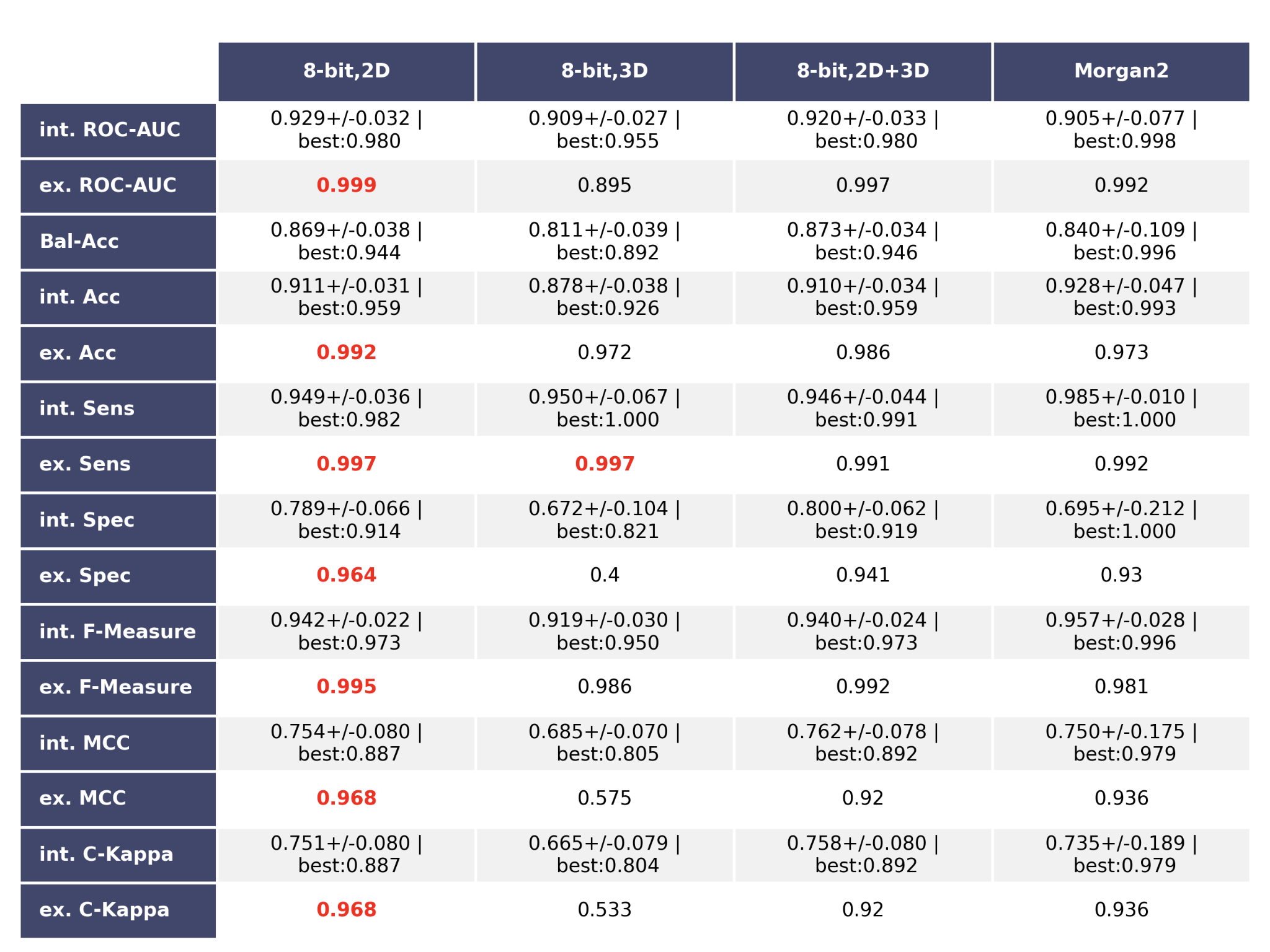
**

**Table S6: Results of RBF SVM and Mahanabolis distance**

**
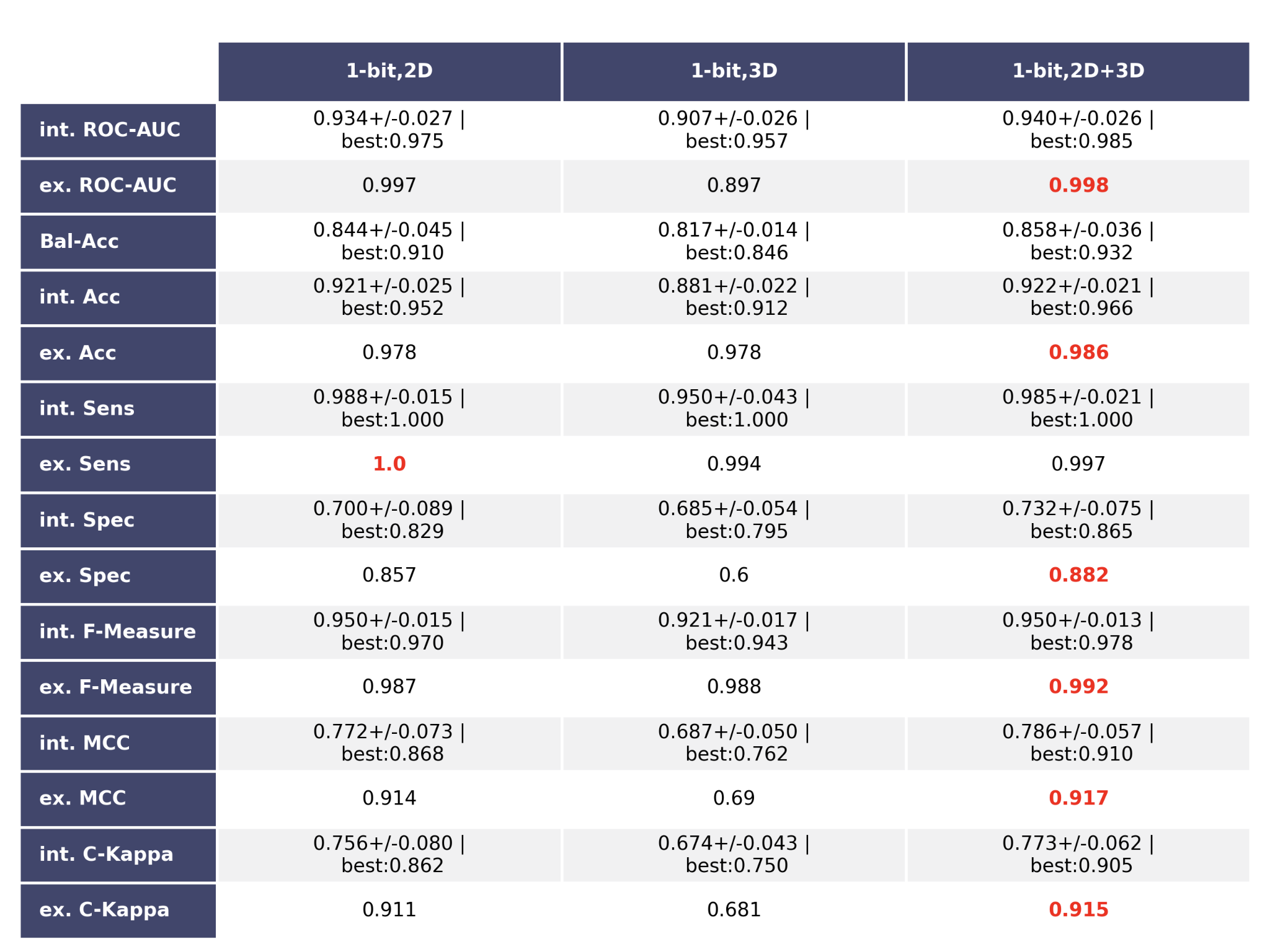
**

**
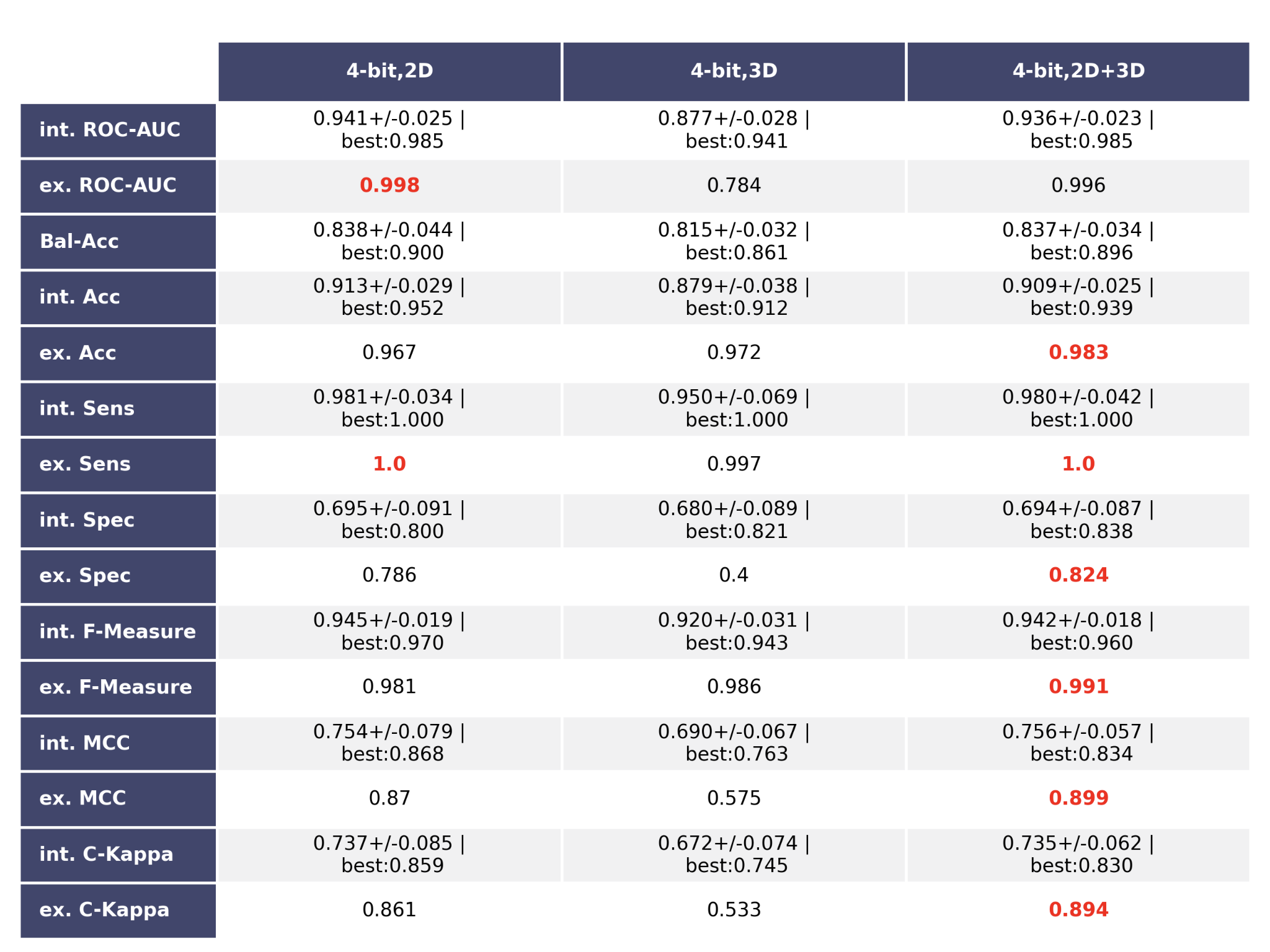
**

**
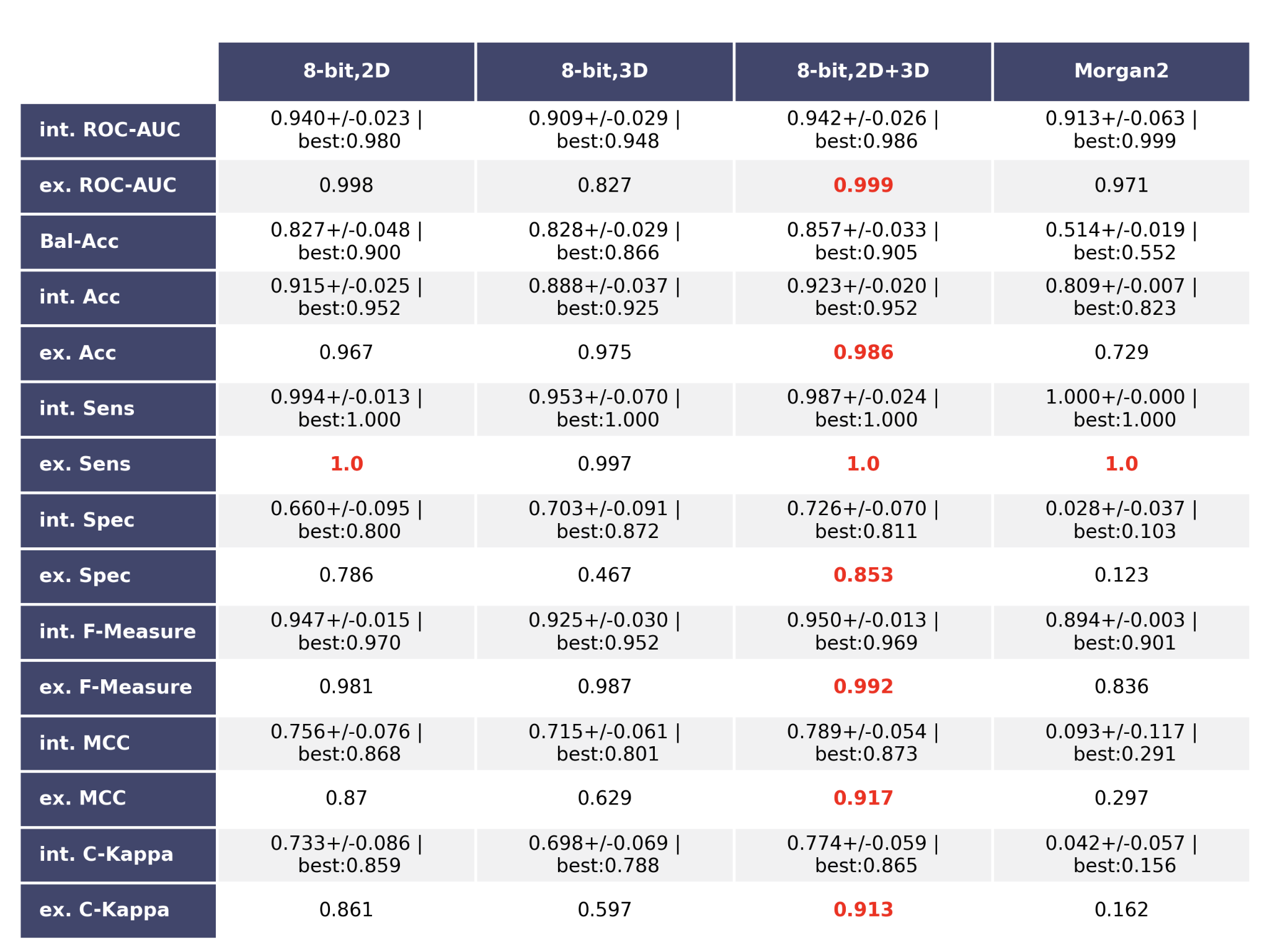
**

**
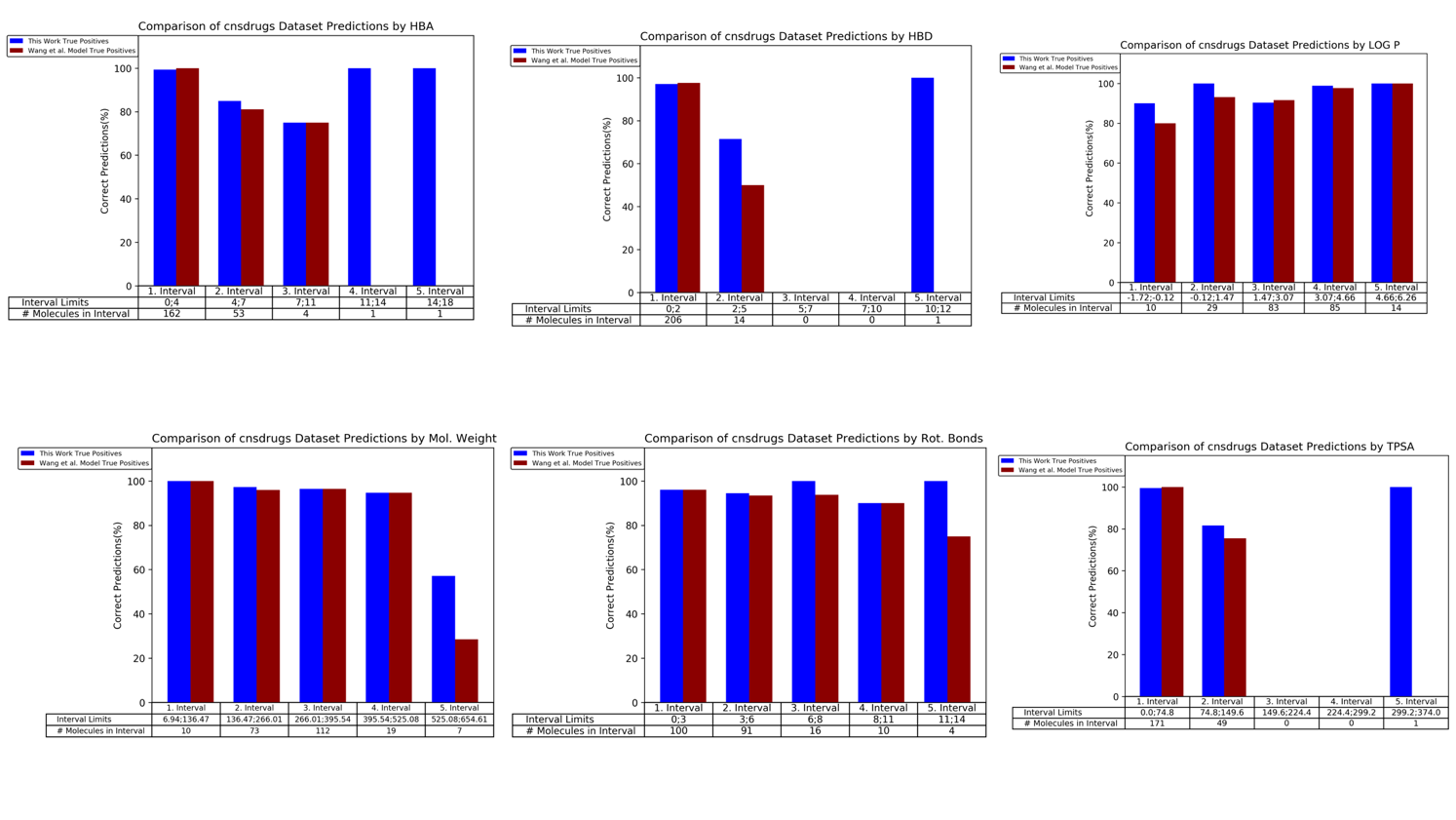
**

**Figure S7:** Shown are the correct predictions (in percent) per bin of the respective descriptor’s value range for both the best model of this work and the webserver of Wang et al.^15^. As shown, our model bbbPythoN-imb (blue) performed well for different intervals as compared to the published model (red).

**
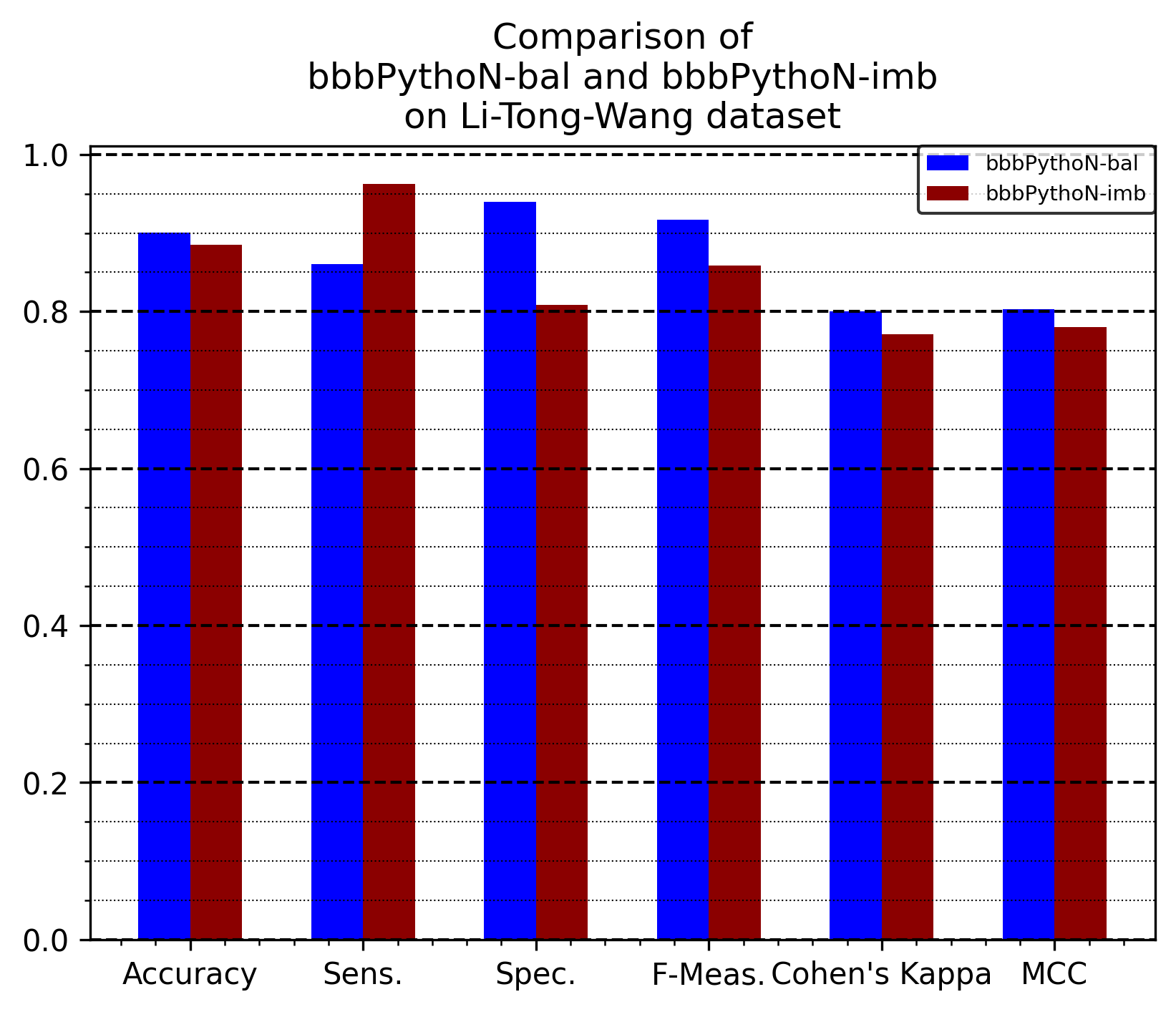
**

**Figure S8:** Comparison of predictive performance on the external validation dataset compiled from Li et al.^52^, Wang et al. ^15^., and Tong et al.^54^ of bbbPythoN-bal and bbbPythoN-imb.

**Table S9: The distribution of active and inactive class for both training and independent test set used in this study.**

|  | **Training set** | **Active/inactive ratio** | **Independent test** | **Active/inactive ratio** |
| --- | --- | --- | --- | --- |
| **2D, Euclidean** | 1474 | 1100/374 | 368 | 339/29 |
| **3D, Euclidean** | 1474 | 1089/385 | 359 | 344/15 |
| **2D+3D, Euclidean** | 1474 | 1099/375 | 359 | 334/25 |
| **2D, Mahalanobis** | 1474 | 1127/347 | 368 | 312/56 |
| **3D, Mahalanobis** | 1474 | 1089/385 | 359 | 344/15 |
| **2D+3D, Mahalanobis** | 1474 | 1108/366 | 359 | 325/34 |
| **Morgan2** | 1474 | 1185/289 | 369 | 255/114 |
| **2D+3D, Mahalanobis,**  **Balanced** | 3291 | 1537/1754 | 765 | 479/286 |


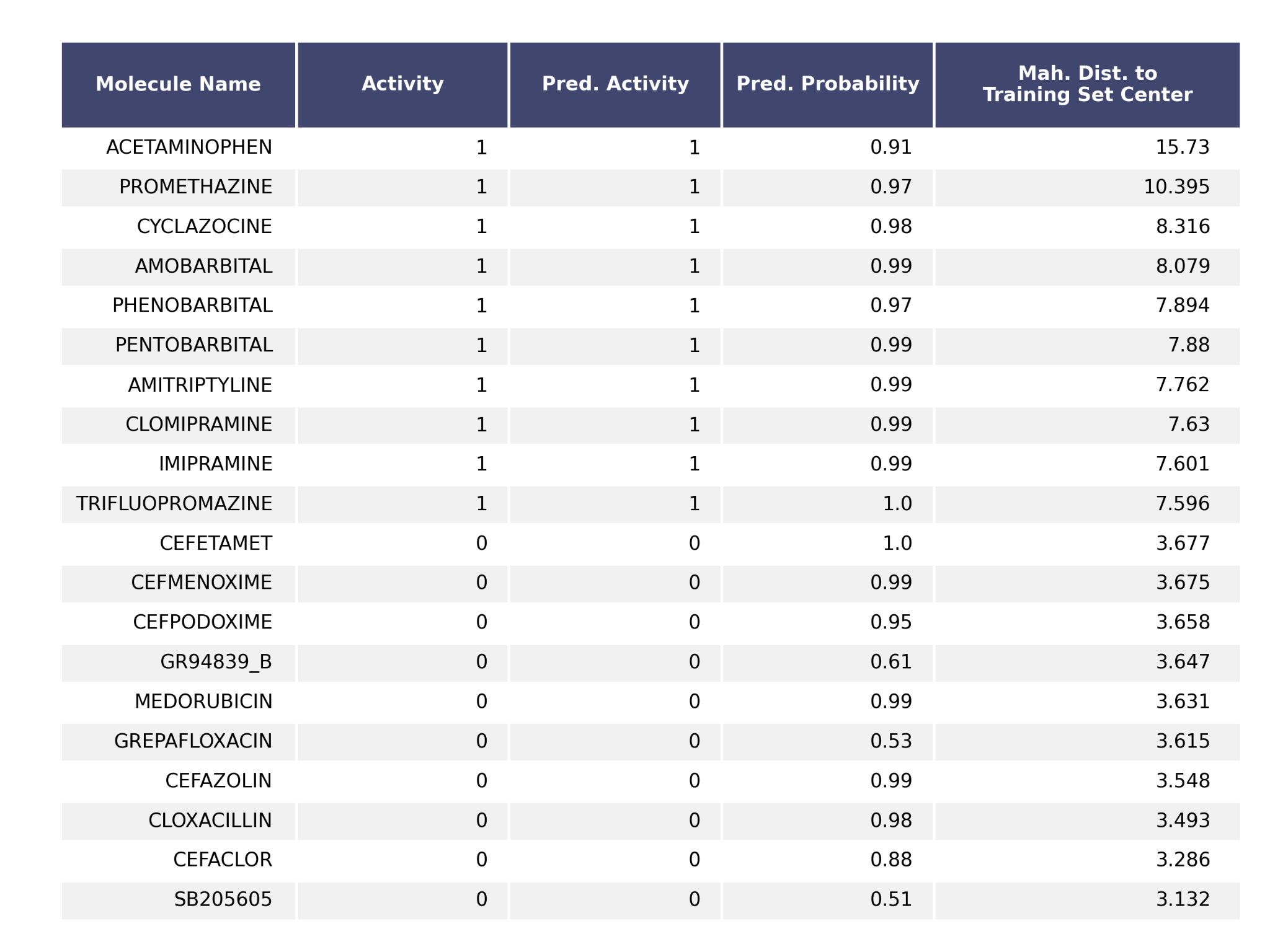


**Table S10:** Twenty test set compounds closest and farthest away from the training set center, ten compounds each, measured in Mahalanobis distance. Activity Predictions and Prediction Probability produced by best performing imbalanced model (Random Forest using 2D+3D, 8-bit descriptor-based MI-DSE Fingerprints trained and tested on Mahalanobis Distance Kennard-Stone training and test sets).


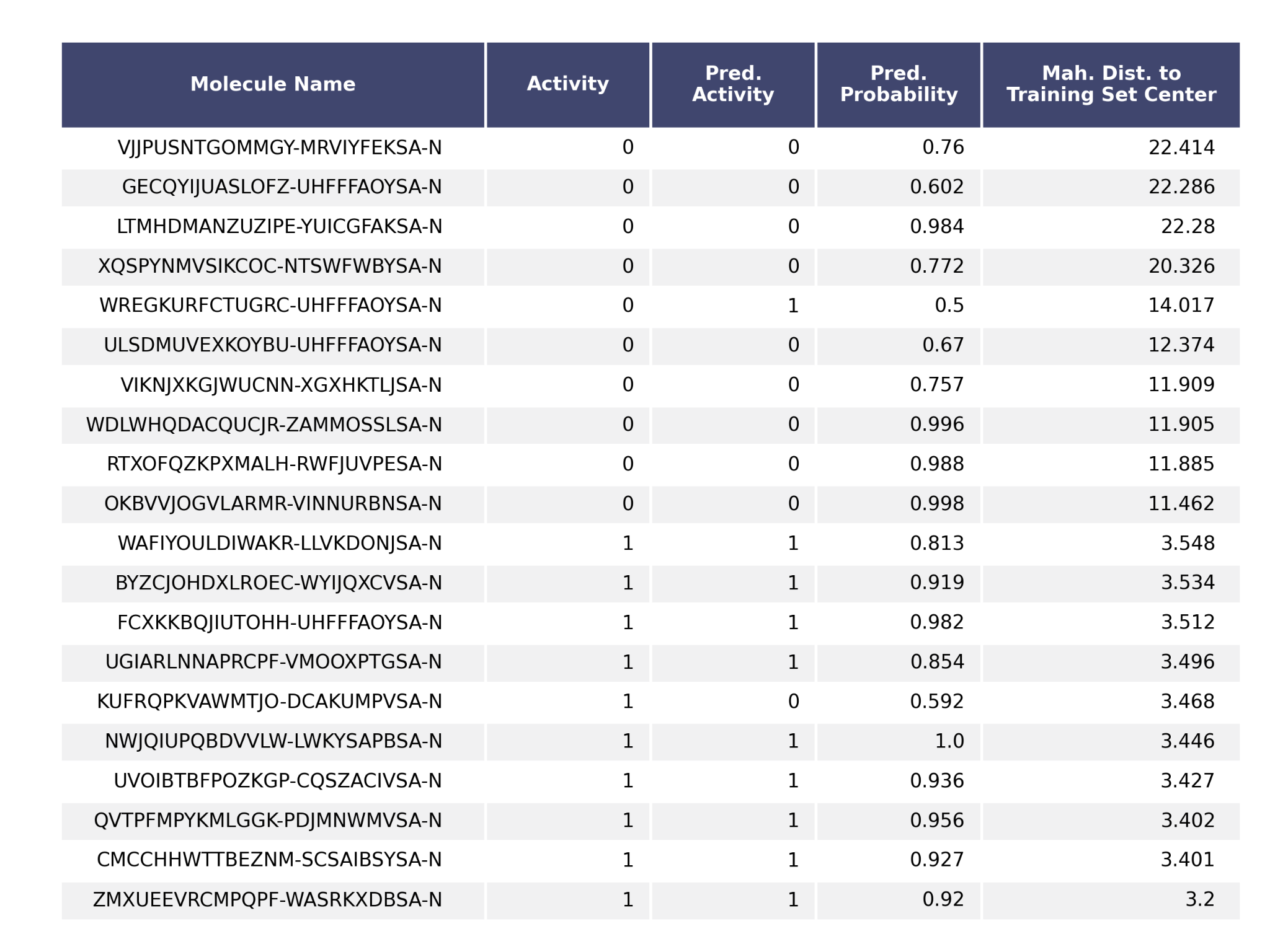


**Table S11:** Twenty test set compounds closest and farthest away from the training set center, ten compounds each, measured in Mahalanobis distance. Activity Predictions and Prediction Probability produced by best performing balanced model (Random Forest using 2D+3D, 8-bit descriptor-based MI-DSE Fingerprints trained and tested on Mahalanobis Distance Kennard-Stone training and test sets).

**
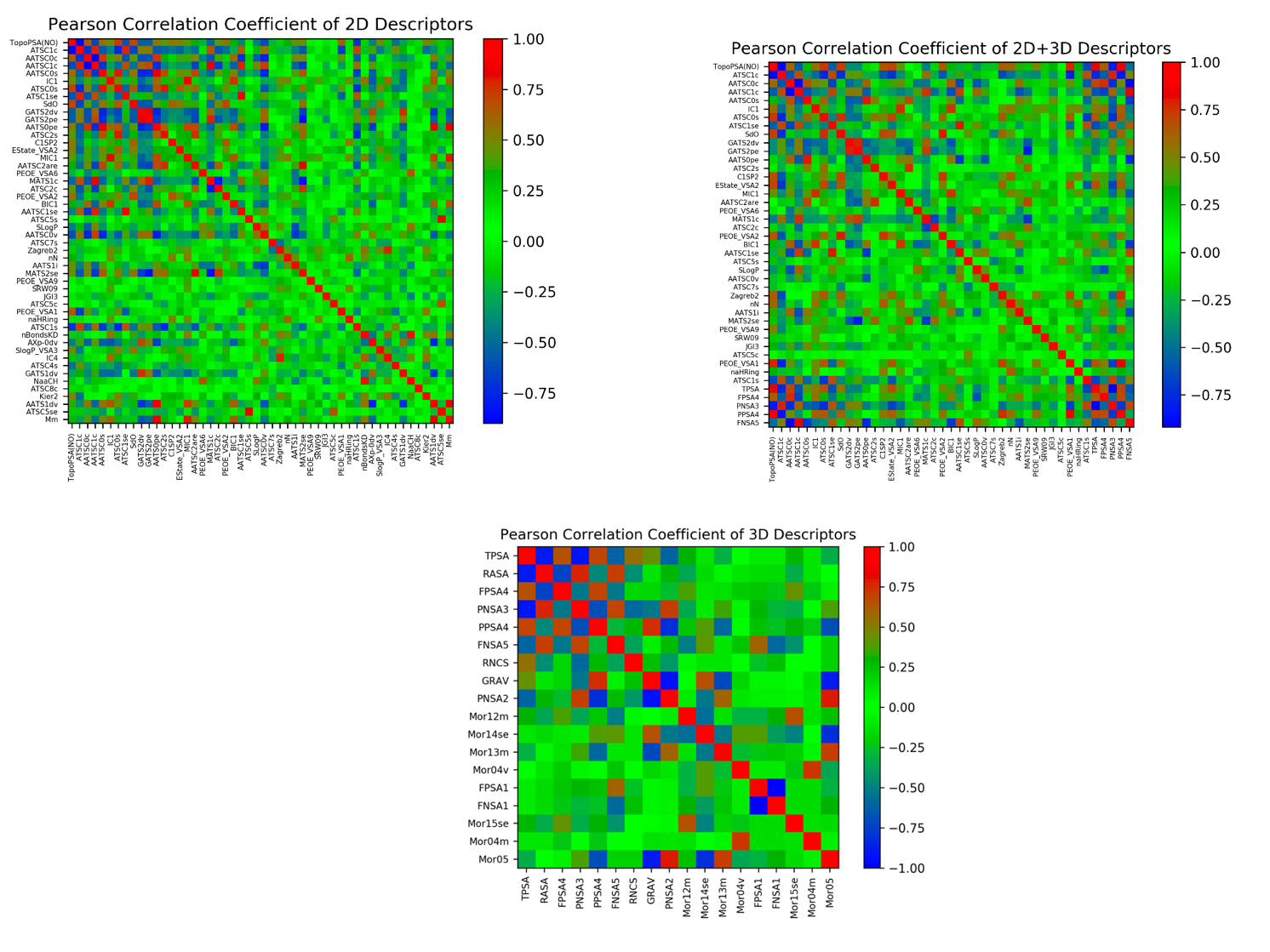
**

**Figure S12:** Pearson Correlation Coefficient analysis of the applied descriptors.

**Table S13:** The 2D and 3D descriptors encoded in the best-performing MI-DSE fingerprint with their respective MI-DSE scores.

| **Descriptor Name (Dimension)** | **MI-DSE Score** |
| --- | --- |
| TopoPSA(NO) (2D) | 0.569 |
| ATSC1c (2D) | 0.319 |
| AATSC0c (2D) | 0.296 |
| IC1 (2D) | 0.293 |
|  |  |
| AATSC0s (2D) | 0.293 |
| ATSC0s (2D) | 0.289 |
| ATSC1se (2D) | 0.285 |
| SdO (2D) | 0.272 |
| GATS2dv (2D) | 0.257 |
| GATS2pe (2D) | 0.249 |
| ATSC2s (2D) | 0.227 |
| AATS0se (2D) | 0.225 |
| EState_VSA2 (2D) | 0.209 |
| MIC1 (2D) | 0.208 |
| BIC1 (2D) | 0.198 |
| ATSC2c (2D) | 0.195 |
| VSA_EState3 (2D) | 0.195 |
| PEOE_VSA6 (2D) | 0.191 |
| AATSC2are (2D) | 0.187 |
| AATSC0v (2D) | 0.187 |
| PEOE_VSA2 (2D) | 0.186 |
| ATSC5s (2D) | 0.182 |
| ATSC7s (2D) | 0.179 |
| AATS1i (2D) | 0.174 |
| nN (2D) | 0.172 |
| Zagreb2 (2D) | 0.169 |
| PEOE_VSA9 (2D) | 0.169 |
| AATSC1c (2D) | 0.163 |
| SRW09 (2D) | 0.162 |
| ATSC5c (2D) | 0.161 |
| PEOE_VSA1 (2D) | 0.159 |
| AATSC1are (2D) | 0.159 |
| MATS2are (2D) | 0.159 |
| naHRing (2D) | 0.151 |
| IC4 (2D) | 0.15 |
| SlogP_VSA3 (2D) | 0.149 |
| CIC2 (2D) | 0.145 |
| AATS1dv (2D) | 0.144 |
| JGI3 (2D) | 0.144 |
| Xch-5d (2D) | 0.143 |
| ATSC4c (2D) | 0.142 |
| ATSC4s (2D) | 0.142 |
| NaaCH (2D) | 0.141 |
| TPSA (3D) | 0.439 |
| PNSA3(3D) | 0.362 |
| FPSA5(3D) | 0.321 |
| DPSA4(3D) | 0.317 |
| FNSA5(3D) | 0.264 |
| RNCS(3D) | 0.154 |

**Table S14:** Structural key features encoded in the best-performing MI-DSE fingerprint.

| Descriptor Name |
| --- |
| MDEO-11 |
| MAXdO |
| MAXsOH |
| MAXdssC |
| MDEN-22 |
| MDEO-12 |
| MAXaaN |
| MDEN-23 |
| MAXaasN |
| MAXaaNH |
| MDEN-12 |
